# Evolution and expression of the phosphodiesterase 6 genes unveils vertebrate novelty to control photosensitivity

**DOI:** 10.1186/s12862-016-0695-z

**Published:** 2016-06-13

**Authors:** David Lagman, Ilkin E. Franzén, Joel Eggert, Dan Larhammar, Xesús M. Abalo

**Affiliations:** Department of Neuroscience, Science for Life Laboratory, Uppsala University, Box 593, SE-75124 Uppsala, Sweden

**Keywords:** Evolution, Genome duplication, Tetraploidisation, Vision, Phototransduction, Teleost, Zebrafish, Retinomotor movements

## Abstract

**Background:**

Phosphodiesterase 6 (PDE6) is a protein complex that hydrolyses cGMP and acts as the effector of the vertebrate phototransduction cascade. The PDE6 holoenzyme consists of catalytic and inhibitory subunits belonging to two unrelated gene families. Rods and cones express distinct genes from both families: *PDE6A* and *PDE6B* code for the catalytic and *PDE6G* the inhibitory subunits in rods while *PDE6C* codes for the catalytic and *PDE6H* the inhibitory subunits in cones. We performed phylogenetic and comparative synteny analyses for both gene families in genomes from a broad range of animals. Furthermore, gene expression was investigated in zebrafish.

**Results:**

We found that both gene families expanded from one to three members in the two rounds of genome doubling (2R) that occurred at the base of vertebrate evolution. The PDE6 inhibitory subunit gene family appears to be unique to vertebrates and expanded further after the teleost-specific genome doubling (3R). We also describe a new family member that originated in 2R and has been lost in amniotes, which we have named *pde6i*. Zebrafish has retained two additional copies of the PDE6 inhibitory subunit genes after 3R that are highly conserved, have high amino acid sequence identity, are coexpressed in the same photoreceptor type as their amniote orthologs and, interestingly, show strikingly different daily oscillation in gene expression levels.

**Conclusions:**

Together, these data suggest specialisation related to the adaptation to different light intensities during the day-night cycle, most likely maintaining the regulatory function of the PDE inhibitory subunits in the phototransduction cascade.

**Electronic supplementary material:**

The online version of this article (doi:10.1186/s12862-016-0695-z) contains supplementary material, which is available to authorized users.

## Background

Phosphodiesterases (PDEs) are a large family of proteins with 21 identified human members divided into 11 subfamilies: PDE1-PDE11 [1]. The present work focuses on the photoreceptor cell-specific PDE6 subfamily, which consist of α, β and α’ catalytic subunits encoded by the *PDE6A*, *PDE6B* and *PDE6C* genes. PDE6 catalytic activity is regulated by the γ and γ’ inhibitory subunits encoded by *PDE6G* and *PDE6H*, respectively [2].

Phylogenetic analyses complemented with chromosomal analyses in human previously published by our lab [3, 4] and phylogenetic data from other investigators [5] suggested that the PDE6 catalytic gene family with the *PDE6A*, *PDE6B* and *PDE6C* genes expanded in the two rounds of whole genome duplications (2R) that occurred early in vertebrate evolution [6, 7]. Other analyses of phylogeny and exon-intron organisation have shown that the PDE6 subfamily is most closely related to the PDE5 and PDE11 subfamilies [5, 8]. 2R was also proposed to explain the duplicates found in the PDE6 inhibitory subunit gene family, whose genes were found to be located in the same paralogon (set of related chromosomes) as the developmentally important homeobox gene clusters [4] that are known to have been duplicated in 2R [9].

PDE6 proteins are expressed in vertebrate rod and cone photoreceptor cells. As for the other components of the phototransduction cascade (see [3] for references), rods and cones use distinct but related PDE6 subunits: rods express the *PDE6A* and *PDE6B* genes, which give rise to a catalytic heterodimer, and the *PDE6G* inhibitory subunit gene, whereas cones express *PDE6C*, resulting in a catalytic homodimer, and the *PDE6H* inhibitory subunit gene [2, 4].

The PDE6 catalytic subunit proteins have two GAF domains (GAF domains are named after the proteins that contain them; cGMP-activated PDEs, adenylyl cyclases and FhlA) followed by one catalytic domain, a structure that is shared with the PDE2, PDE5, PDE10 and PDE11 subfamilies [1]. The catalytic domain is present in all of the different PDE subfamilies, with differences in substrate specificity [1]. The PDE6 enzymes are distinguished from the other PDE subfamilies by a much higher catalytic activity and two accessory inhibitory subunits that interact with a GAF domain and the catalytic domain of the catalytic subunits and thus block activity during dark conditions [1, 10]. The emergence of the inhibitory subunits has been proposed as one of the events that made it possible for a higher catalytic rate to evolve in PDE6 compared to the other PDEs, resulting in a fast photoreceptor response [2].

The PDE6 holoenzyme function can be summarised as follows. The cascade is initiated by an opsin, which is activated by a photon. There are different opsins with distinct spectral selectivity mediating colour vision in various types of cones and a rhodopsin mediating dim-light vision in rods. Activated opsin acts as a G nucleotide exchange factor for the heterotrimeric G-protein transducin. A GTP molecule replaces GDP at the active site of the alpha subunit of transducin leading to dissociation of the transducin heterotrimer into the activated alpha subunit and a heterodimer of the beta and gamma subunits. The alpha transducin then activates the PDE6, a cGMP phosphodiesterase. Activation takes place when transducin alpha subunits remove the two PDE6 inhibitory subunits [11]. The activated PDE6 hydrolyses cGMP into GMP, which reduces the cGMP levels in the cell and leads to a closure of cyclic nucleotide-gated channels and hyperpolarisation of the photoreceptor cell. The regulation of activity by the PDE6 inhibitory subunits involves specific regions within these small proteins responsible for the binding to the PDE6 catalytic subunits and the alpha subunit of transducin but also for the stabilisation of the binding affinity, the stimulation of non-catalytic cGMP binding, the increase in cGMP exchange at the GAF domains and the stimulation of GTPase activity of transducin, in complex with RGS9-1 [12].

In this study, an extensive repertoire of vertebrate species was used to resolve the evolutionary history of the PDE6 subunit gene families and their chromosomal regions more precisely. This gives us a robust dataset, which demonstrates the expansion of both the catalytic and inhibitory subunit gene families in 2R as well as further duplication of the inhibitory subunit gene family in the teleost-specific whole genome duplication (3R) as well as through local duplications. Previous studies have described the same set of catalytic subunit gene repertoire in zebrafish as in human: *pde6a, pde6b* and *pde6c* [13, 14]. Here we demonstrate that zebrafish has retained, as a result of 3R, two paralogous genes for each of the two PDE6 inhibitory genes found in human: *pde6ga*, *pde6gb*, *pde6ha* and *pde6hb*. Additionally, we also show that zebrafish has retained an extra PDE6 inhibitory subunit gene from 2R that we named *pde6i* which is not present in amniotes. Due to the importance of zebrafish as a model for both evolution and visual function and disorders, we have analysed the expression of all PDE6 genes in zebrafish and found striking differences related to the adaptation to different light intensities during the day-night cycle.

## Results

### Three PDE6 catalytic subunit genes expanded in 2R and were subsequently retained in most vertebrate lineages, with minimal to no expansion in 3R

**Fig. 1:**
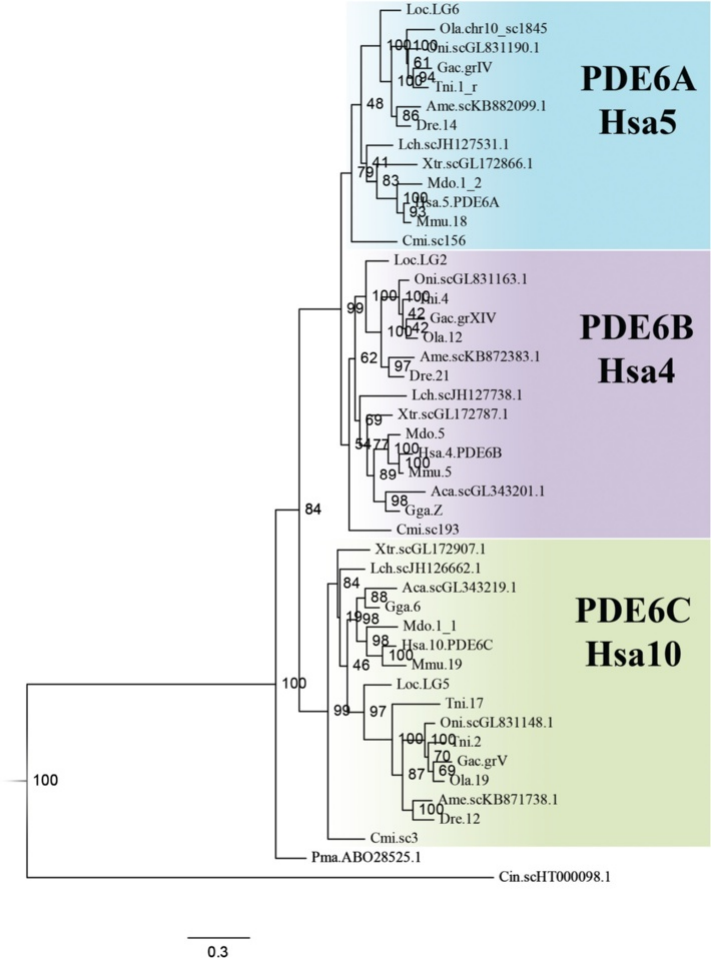
Phylogenetic tree of the vertebrate PDE6 catalytic subunit genes. The phylogenetic maximum likelihood method was used to obtain the tree, rooted with the human *PDE5A* and *PDE11A* sequences. The three-letter abbreviations represent species and the number or Roman numeral represents chromosome, linkage group, scaffold, contig or NCBI accession number. The coloured boxes include the sequences that cluster together with the three different human PDE6 catalytic subunit genes. A more detailed PhyML tree with regard to birds and non-avian reptiles are presented in Additional file 3: Figure S1

An alignment of the PDE6 catalytic subunit amino acid sequences used in the phylogenetic maximum likelihood (PhyML) analysis is provided in Additional file 1 and the sequence identifiers and the genomic locations of the included sequences are provided in Additional file 2. The PhyML tree, rooted with the human *PDE5A* and *PDE11A* genes, of this family forms three well-supported clades with each of the human *PDE6A, PDE6B* and *PDE6C* genes and their orthologues (Fig. 1). All vertebrate genomes investigated have three PDE6 catalytic subunit genes, with a few exceptions. The first exception is the green spotted pufferfish, which has two *PDE6C* paralogs; one located on chromosome 17, that clusters basally to the other teleost sequences, and the other on chromosome 2, that clusters together with the Nile tilapia, three-spined stickleback and medaka sequences. Interestingly, two *PDE6C* genes were also found in the fugu genome (data not shown), indicating either a pufferfish-specific duplication (with rapid evolution of the duplicate) or that the pufferfishes have retained 3R duplicates that have been lost in other teleost fish, a scenario supported by the phylogenetic analysis (Fig. 1). The second exception is the absence of the *PDE6A* gene in birds and non-avian reptiles (Additional file 3: Figure S1).

We identified a single gene in the arctic lamprey (*Lethenteron camtschaticum*) genome assembly (data not shown). This gene has previously been identified in the sea lamprey [5], indicating that northern hemisphere lampreys have retained only one catalytic subunit gene. The sea lamprey sequence clusters basally to all other PDE6 catalytic subunit clusters in the phylogenetic analysis (Fig. 1), thus we cannot confidently assign orthology to any of the gnathostome sequences. The invertebrate chordate *Ciona intestinalis* has one gene that has been assigned the same Ensembl protein family ID as the vertebrate PDE6 catalytic subunit genes. This sequence was added to the analysis for relative dating of the duplications of the vertebrate genes and it clusters basally to the three vertebrate clusters, suggesting that it is ortholog to the ancestor of the vertebrate *PDE6A, PDE6B* and *PDE6C* genes. This is in agreement with the previously suggested expansion of this gene family in 2R, based on much fewer sequences [3, 4].

### The chromosomal region housing the PDE6 catalytic subunit genes belongs to a paralogon that arose in 2R

The human *PDE6A, PDE6B* and *PDE6C* genes are located on chromosomes 5, 4 and 10, respectively, like the well-studied neuropeptide Y receptor genes and many neighbouring gene families. These have been previously demonstrated to have expanded in the early vertebrate tetraploidisations [15]. As the PDE6 catalytic subunit genes are located some distance away from those reported previously, we analysed additional families in these regions to see whether the duplications could have taken place in the timeframe of 2R.

A total of eleven neighbouring gene families were identified as having members in the same chromosomal regions as the three PDE6 catalytic subunit genes (Additional file 3: Table S1 and Additional file 2). Three of the neighbouring gene families (CP, SLC26A and ZNF) were excluded at an initial stage of the analysis due to their complex phylogenetic tree topology or their multitude of members. For the remaining eight families four have annotated invertebrate and vertebrate members, DPYS, PPP2R2, STK32 and TBC1D; while the other four, ABLIM, AFAP, JAKMIP and SH3TC, only have vertebrate members. The sequence identifiers and genomic locations of the included neighbouring gene families are provided in Additional file 2.

The phylogenetic trees and the species representation of the STK32 gene family support an expansion in 2R giving rise to three vertebrate genes (Additional file 3: Figure S2). However, teleost fish appear to have lost the *STK32B*. Early origin of this gene is shown by its presence outside tetrapods, in the genomes of the coelacanth and the spotted gar. The phylogenetic tree of the TBC1D family shows two clusters consistent with duplication in the same timeframe as 2R (Additional file 3: Figure S3). The PPP2R2 phylogenetic tree supports an expansion in 2R and a possible expansion of the *PPP2R2A* genes in the teleost specific 3R event (Additional file 3: Figure S4). The phylogenetic tree of the DPYS family shows six gene clusters, four of which, namely *CRMP1, DPYSL2, DPYSL3* and *DPYSL4*, seem to form a vertebrate subfamily clustering with a basal putative *Ciona intestinalis* ortholog (Additional file 3: Figure S5). This is consistent with an expansion in 2R. The topology also supports a duplication of the teleost *DPYSL2* genes in 3R. The two other gene clusters that include the human *DPYS* and *DPYSL5* genes with their vertebrate homologs seem to have branched off from the previously mentioned vertebrate subfamily before the divergence of the lineages leading to tunicates and vertebrates.

The phylogenies of the ABLIM, AFAP, JAKMIP and SH3TC families show that these families expanded before the separation of lobe-finned and ray-finned vertebrates (Additional file 3: Figures S6-S9). However, if this expansion occurred due to 2R is not clear due to the lack of invertebrate family members for relative dating. The ABLIM family shows evidence for a duplication of both the *ABLIM1* gene and the *ABLIM3* gene in 3R (Additional file 3: Figure S6).

**Fig. 2:**
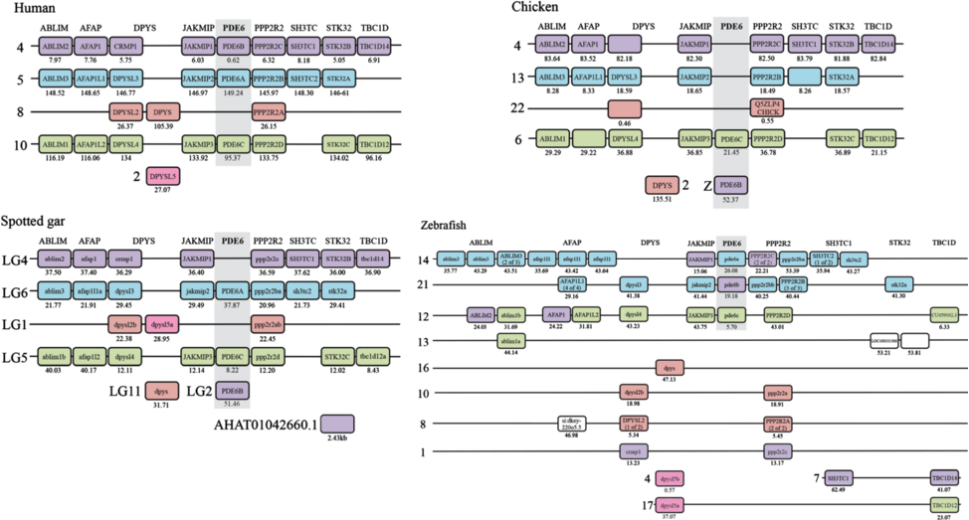
Conserved synteny of the chromosomal blocks carrying the PDE6 catalytic subunit genes resulting from 2R. The figure illustrate the identified chromosomal regions harbouring the PDE6 catalytic subunit gene family and the neighbouring gene families in human, chicken, spotted gar and zebrafish. The presence of a quartet of regions in human, chicken and spotted gar is consistent with 2R expansion resulting in a paralogon. In the zebrafish the chromosomal regions are rearranged which is consistent with chromosomal rearrangements after 3R in the teleost lineage. The families are shown in alphabetical order and each of the boxes are coloured after the chromosomal location of their human ortholog. The numbers under the boxes are the genomic locations in mega base pairs unless otherwise stated

A summary of the localisation of the identified neighbouring gene family genes as well as the PDE6 catalytic subunit genes on human, chicken, spotted gar and zebrafish chromosomes are presented in Fig. 2.

### An ancestral PDE6 inhibitory subunit gene emerged in the vertebrate ancestor and duplicated in 2R and 3R

An amino acid sequence alignment of the identified PDE6 inhibitory subunit genes is provided in Additional file 4 and the sequence identifiers and genomic locations for the included sequences are listed in Additional file 2. Due to the relatively high level of sequence conservation, 72–81 % between lampreys and humans (data not shown), ≥64 % between human and zebrafish (see Table 1) and short sequence lengths, no reliable phylogenetic signal can be obtained for the PDE6 inhibitory subunit genes. Therefore, their chromosomal locations were thoroughly investigated and used for assigning orthology. As a result, we discovered that this gene family has three paralogs (*PDE6G, PDE6H* and *PDE6I*) that are located in the same chromosomal regions as the somatostatin receptor 2, 3 and 5 genes (*SSTR2, SSTR3* and *SSTR5*) and the urotensin receptor genes, previously shown to reside in a paralogon [16, 17]. Thus, now when more genomic resources are available we can show that the PDE6 inhibitory subunit genes are located in a different paralogon than the previously suggested *HOX* paralogon [4].

**Table 1:**
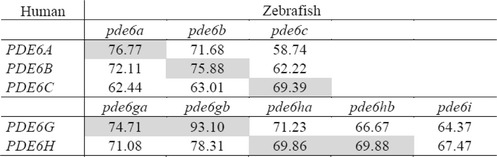
Amino acid sequence identity between human and zebrafish PDE6 subunits

Percentage of identity between human and zebrafish PDE6 amino acid sequences. A comparison between the human and the zebrafish PDE6 amino acid sequences was done in order to investigate their conservation. The calculations were done using pair-wise alignments in JalView 2.7. Shaded boxes are comparisons between orthologs

**Fig. 3:**
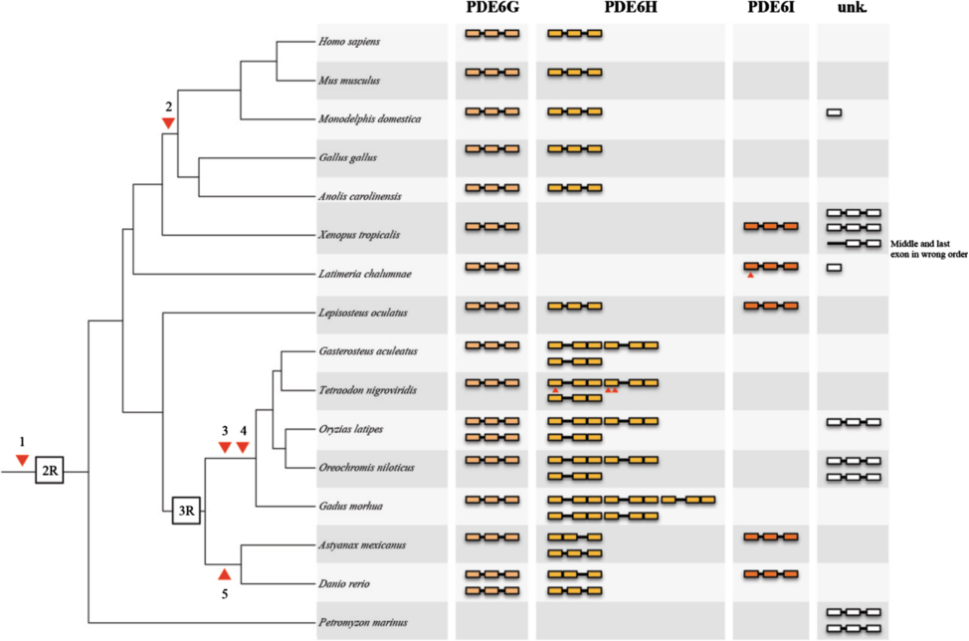
Proposed evolutionary history and gene repertoire of the PDE6 inhibitory subunit genes. Important events in the evolution of this gene family 1) Emergence of the ancestral PDE6 inhibitory subunit gene, before 2R. 2) Loss of *PDE6I* in the ancestor of amniotes. 3) Loss of the second intron of the coding region of the putative *PDE6H* 3R duplicates. 4) Local duplication of one of the *PDE6H* 3R duplicates. 5) Loss of the first intron of the coding region in one of the *PDE6H* 3R duplicates. Small arrowheads represent stop codons or frame shift mutations in the coding sequence. The orthology assignment of the genes was done according to conservation of synteny and exon-intro organisation. The column to the right shows identified genes whose assignment was not possible

Previously, only *PDE6G* and *PDE6H* have been described in different vertebrate species, thus our identification of *PDE6I* reveals that this family has a third member (Fig. 3). The three PDE6 inhibitory subunit genes were found on linkage groups (LG) 10, 12 and 13 of the spotted gar genome, which also carries *SSTR2*, *SSTR3* and *SSTR5* genes, respectively. A recent analysis of the eye transcriptome of the Florida gar (*Lepisosteus platyrhincus*) showed that the *PDE6I* is expressed (T.D. Lamb et al., personal communication).

The coelacanth has three different PDE6 inhibitory subunit genes in its genome assembly, one fragmented (of unknown orthology, located on scaffold JH132110.1), one full length (*PDE6G* located on scaffold JH126581.1) and one that appears to be a pseudogene due to a frame-shift mutation in the first exon disrupting the translation (*PDE6I* located on scaffold JH127264.1) (Fig. 3 and Additional file 3: Figure S10). The Western clawed frog has four full-length and one fragmented PDE6 inhibitory subunit genes all located on different genomic scaffolds. We could confidently assign the identity as *PDE6G* and *PDE6I* for two of the frog genes (located on scaffolds GL172940.1 and GL172810.1 respectively), whereas the others did not share synteny with any of the spotted gar chromosomal regions carrying a PDE6 inhibitory subunit gene (Fig. 3 and Additional file 3: Figure S10).

We identified orthologs for *PDE6G* and *PDE6H* in the teleost species investigated but only zebrafish and Mexican cave tetra has retained *PDE6I* (Additional file 3: Figures S11-S12 and Additional file 5). Most of the teleost species investigated have retained putative 3R duplicates for *PDE6H*, while only zebrafish and medaka have retained 3R paralogs for *PDE6G* (*pde6ga* and *pde6gb*) (Fig. 3). The teleost *PDE6H* genes have experienced intron loss at different time points during teleost evolution. The zebrafish and Mexican cave tetra have lost the first intron of the coding region in the *pde6ha* gene (located on chromosome 6 in zebrafish), thus it probably took place in their common ancestor. The other investigated teleosts have lost the last intron of the coding region in their *PDE6H* genes (see Fig. 3). These intron losses along with the conservation of synteny allowed assignment of orthology of the teleost PDE6 inhibitory subunit genes. For the complete repertoire of the PDE6 inhibitory subunit genes identified in teleost genomes see Fig. 3. For synteny comparisons of teleost scaffolds or chromosomes with PDE6 inhibitory subunit genes to spotted gar linkage groups, see Additional file 3: Figures S11-S17.

The *PDE6I* gene could not be identified in the elephant shark (tentatively only *PDE6G* and *PDE6H*) or in amniotes. In chicken, the *PDE6G* and *PDE6H* genes are located close to the *SSTR2* gene on chromosome 18 and *SSTR3* on chromosome 1, respectively. The human *PDE6G* gene is located on chromosome 17 as is the *SSTR2* gene. In contrast, the human *PDE6H* gene on chromosome 12 is not located on the same chromosome as the *SSTR3* gene, which is located on chromosome 22 (Additional file 5). However, the opossum *PDE6H* gene is located on the same chromosome as the *SSTR3* gene, chromosome 8, which suggests that a translocation of *PDE6H* took place in the primate lineage.

Lampreys are a special case. Two PDE6 inhibitory subunit sequences have previously been cloned in the sea lamprey: GenBank accession number ABO16480.2 and NCBI accession number ABO64650.1 [5]. Due to the lack of synteny data we could not assign their orthology to the other vertebrate genes (Fig. 3). In the arctic lamprey genome we could only identify sequence fragments: one first exon, one middle exon and three last exons, all located on different scaffolds or contigs that prevented us to assign any orthology.

In order to analyse the evolutionary origin of these genes, we performed TBLASTN searches in all invertebrate genomes present in the NCBI whole genome shotgun reference database, using the human *PDE6G* and *PDE6H* amino acid sequences as templates. We also performed *nhmmer* searches in the invertebrate genomes present in Table 2. Despite these efforts, not a single homologous sequence for the PDE6 inhibitory subunit genes was identified in invertebrates.

**Table 2:** List of the species used in this study

Species; scientific name (abbreviation in trees)	Assembly
Acorn worm; *Saccoglossus kowalevskii* (Sko)^a^	Skow_1.1
Fruitfly; *Drosophila melanogaster* (Dme)	BDGP5
Lancelet; *Branchiostoma floridae* (Bfl)^a^	v2.0
Lancelet (transcriptome); *Branchiostoma lanceolatum* (Bla)^a^	PRJNA82409
Pelagic tunicate; *Oikopleura dioica* (Odi)^a^	ASM20953v1
Purple sea urchin; *Strongylocentrotus purpuratus* (Spu)^a^	Spu_3.1
Vase tunicate; *Ciona intestinalis* (Cin)^a^	JGI2/KH
Arctic lamprey; *Lethenteron camtschaticum* (Lca)	LetJap1.0
Sea lamprey; *Petromyzon marinus* (Pma)	Pmarinus_7.0
Elephant shark; *Callorhinchus milii* (Cmi)	Callorhinchus_milii-6.1.3
Spotted gar; *Lepisosteus oculatus* (Loc)	LepOcu1
Coelacanth; *Latimeria chalumnae* (Lch)	LatCha1
Atlantic cod; *Gadus morhua* (Gmo)	gadMor1
Green spotted pufferfish; *Tetraodon nigroviridis* (Tni)	TETRAODON 8.0
Fugu; *Takifugu rubripes* (Tru)	FUGU 4.0
Medaka; *Oryzias latipes* (Ola)	HdrR
Mexican cave tetra; *Astyanax mexicanus* (Ame)	AstMex102
Nile tilapia; *Oreochromis niloticus* (Oni)	Orenil1.0
Three-spined stickleback; *Gasterosteus aculeatus* (Gac)	BROAD S1
Zebrafish; *Danio rerio* (Dre)	Zv9
Western clawed frog; *Xenopus tropicalis* (Xtr)	JGI_4.2
Grey short tailed opossum; *Monodelphis domestica* (Mdo)	monDom5
Human; *Homo sapiens* (Hsa)	GRCh37
Mouse; *Mus musculus* (Mmu)	NCBI m37/GRCm38
American alligator; *Alligator mississippiensis* (Ami)^b^	allMis0.2
Brumese python; *Python molurus bivittatus* (Pmo)^b^	Python_molurus_bivittatus-5.0.2
Chinese alligator; *Alligator sinensis* (Asi)^b^	ASM45574v1
Chinese softshell turtle; *Pelodiscus sinensis* (Psi)^b^	PelSin_1.0
Green anole lizard; *Anolis carolinensis* (Aca)	AnoCar2.0
Green sea turtle; *Chelonia mydas* (Cmy)^b^	CheMyd_1.0
Painted turtle; *Chrysemys picta bellii* (Cpi)^b^	ChrPicBel3.0.1
Chicken; *Gallus gallus* (Gga)	WASHUC2
Collared flycatcher; *Ficedula albicollis* (Fal) 2	FicAlb_1.4
Mallard; *Anas platyrhynchos* (Apl)^b^	BGI_duck_1.0
Ostrich; *Struthio camelus australis* (Sca)^b^	ASM69896v1
Zebra finch; *Taeniopygia guttata* (Tgu)^b^	taeGut3.2.4

^a)^ Included invertebrate species for *nhmmer* searches for PDE6 inhibitory subunit genes

^b)^ Additional bird and non-avian reptiles included in the searches for *PDE6A* (Additional file 3: Figure S1)

List and details of the species used in this study. The current table shows 5 the common name, scientific name abbreviation and source for all species used to 6 perform the phylogenetic and analyses of conserved synteny

### Comparison of zebrafish PDE6 inhibitory subunit paralog sequences reveal highly conserved regions

Using zebrafish as a model, we investigated the possible specialisations of the PDE6 subunit genes; *pde6a*, *pde6b* and *pde6c* for the catalytic subunits and the PDE6 inhibitory subunit genes retained after 3R; *pde6ga*, *pde6gb*, *pde6ha* and *pde6hb*. A comparison of the amino acid sequence identity between human and zebrafish for the PDE6 inhibitory subunits confirmed high level of conservation: human *PDE6G* shares 75 % and 93 % identity with zebrafish *pde6ga* and *pde6gb*, respectively; while human *PDE6H* displays approximately 70 % identity with both zebrafish *pde6ha* and *pde6hb* (Table 1).

**Fig. 4:**
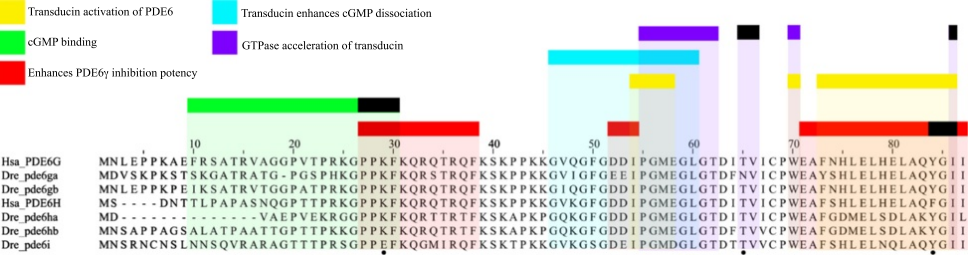
Alignment of the human and zebrafish PDE6 inhibitory subunit amino acid sequences. The alignment was made using ClustalWS and manually curated. Black boxes represent the residues necessary for the function indicated by the different colours. Information about the function of different regions was retrieved from Fig. 7 in [12]

Additionally, an amino acid sequence alignment of the human and zebrafish PDE6 inhibitory subunit sequences showed some differences in each gene, but the regions that are known to be involved in specific functions, previously described in [12], are highly conserved (Fig. 4). Some exceptions were observed, like an asparagine instead of threonine at position 65 in *pde6ga*, a phenylalanine instead of a tyrosine at position 84 in human *PDE6H* and one alteration that could have a functional effect in the *pde6i* carrying a glutamic acid instead of the lysine at position 29 (Fig. 4). These results, together with the gene expression data provided in the sections that follow, suggest that the specialisations did not involve any neofunctionalisation at the protein structure level.

The percentage of identity for the 3′UTR sequences of the zebrafish PDE6 inhibitory subunit genes were analysed in order to find regions suitable for use in the design of probes able to distinguish between the 3R duplicates. We found that, *pde6ga* and *pde6gb* share 48 % nucleotide sequence identity, *pde6ha* and *pde6hb* share 59 %, and *pde6i* shares between 48–52 % identity with the others (Additional file 3: Table S2).

### The zebrafish PDE6 inhibitory subunit gene paralogs are expressed in the same cell types as their amniote orthologs

The expression pattern of the three PDE6 catalytic subunit genes present in zebrafish*,* has been previously reported [13]. In this investigation, we have analysed the expression levels for all zebrafish PDE6 genes by qRT-PCR, as well as the expression pattern of the PDE6 inhibitory subunit genes; *pde6ga*, *pde6gb*, *pde6ha*, *pde6hb* and *pde6i*, by in situ hybridisation (ISH). *Pde6i* was excluded from the ISH experiments due to the extremely low expression levels found in adult eyes (see next section).

**Fig. 5:**
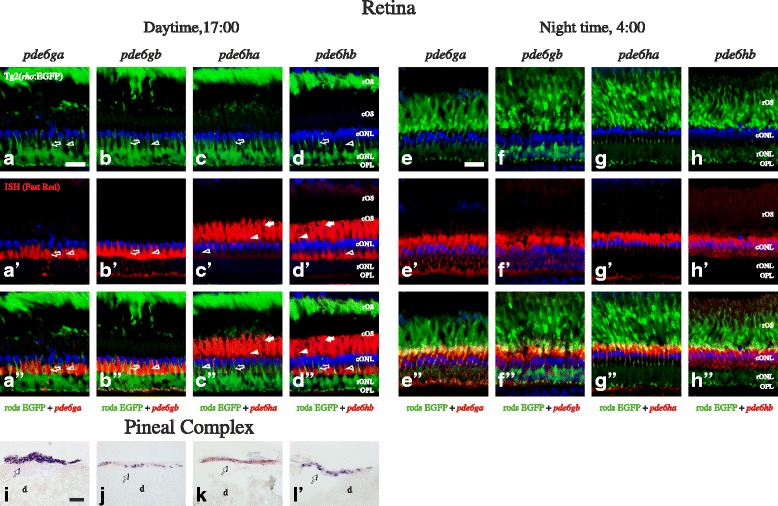
Expression pattern of the PDE6 inhibitory subunit genes in the adult zebrafish retina and pineal complex. Photomicrographs from ISH experiments on adult Tg2(*rho*:EGFP) zebrafish showing staining for *pde6ga*, *pde6bg*, *pde6ha* and *pde6hb* in the retina at 17:00 PM (**a**-**d´´**) and at 4:00 AM (**e**-**h´´**), and also in the pineal complex (**i**-**l**). The upper row shows EFGP fluorescence (green) in rods (**a**-**h**), the second row from the top shows the ISH experiments stained with Fast Red (red), (**a´**-**h´**), the second from the bottom is a merge of the two previous (**a´´**-**h´´**) and the bottom row shows ISH stained with NBT/BCIP (**i**-**l**). DAPI, in blue, stains cell nuclei in all fluorescent photomicrographs. Stricken morphological differences, due to the retinomotor movements, can be observed in the retina between day and night, graphically represented by the contraction of the rOS from the outermost layer at daytime (**a**-**d**) to inner locations at night (**e**-**h**). This makes very difficult the cell assignment in the night retinae, so it was shown only in the day retinae (**a**-**d´´**). Staining for *pde6ga* (**a´**-**a´´** and **e´**-**e´´**) and *pde6gb* (**b´**-**b´´** and **f´**-**f´´**) can be observed in rod inner segments and for *pde6ha* (**c´**-**c´´** and **g´**-**g´´**) and *pde6hb* (**d´**-**d´´** and **h´**-**h´´**) in cones. Staining for all four genes was observed in adult pineal complex (**i**-**l**). In **a´**-**a´´** and **b´**-**b´´**, staining in the SSC seems to be present, therefore, we cannot discard that *pde6ga* and *pde6gb* are expressed in rods and SSC. In addition, the yellowish colour observed in 5**g´´** could suggest double staining; however its absence in the day retina, combined with the about 100 fold lower expression levels and the loose morphology of the night retinae led us to conclude that this is just an artefact. Abbreviations: d; diencephalon, cONL; cone outer nuclear layer, cOS; cone outer segments, OPL; outer plexiform layer, rONL; rod outer nuclear layer, rOS; rod outer segments. Arrows point at DC, arrowheads to LSC, empty arrowheads to SSC and empty arrows to rods´ myoids. In **i**-**l**, arrows point at the pineal complex. Scale bars; in **a** is 20 μm for **a**-**d´´**, in **e** is 20 μm for **e**-**h´´** and in **i** is 30 μm for **i**-**l**

**Fig. 6:**
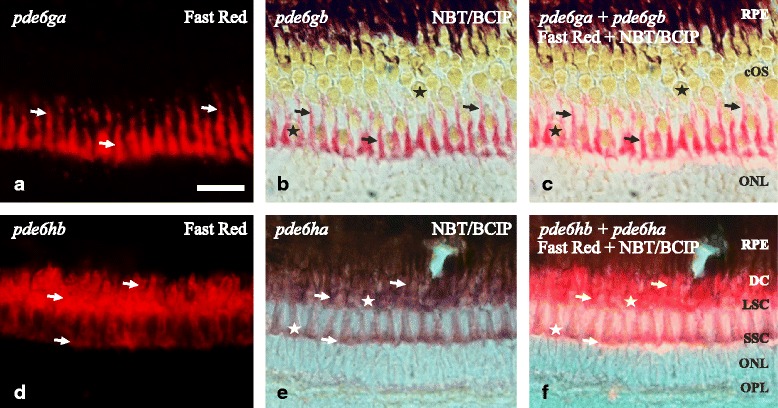
Coexpression in AB zebrafish retina of the 3R generated PDE6 inhibitory subunit paralogs. Photomicrographs from double ISH experiments on adult zebrafish outer retinae show colocalisation for *pde6ga* and *pde6gb* in rods (**a**-**c**) and for *pde6ha* and *pde6hb* in cones (**d**-**f**), arrows are pointing at the same cell in the three corresponding pictures. **a** and **d** are fluorescent pictures from Fast Red staining and **b** and **e** are bright-field pictures from, mainly, NBT/BCIP staining in purple but also a minimal staining from the Fast Red. **c** and **f** combine bright-field and fluorescence to show co-staining in the same cells. **a**-**c** shows exclusive staining in the myoids of the rods. The sections were obtained in an oblique angle to facilitate the perception of the rods´ myoids “crawling” between the cones, despite giving an unreal perception of the stratification. **d**-**f** show staining in the three cone types: SSC, LSC and DC. However, the tight packing of the DC makes it difficult to visualise them with fluorescence (**d**, **f**). Stars mark the cone-specific ellipsoids. Scale bar is 25 μm. RPE; retinal pigment epithelium. For the rest of abbreviations see Fig. 5 legend

There is a strikingly different morphology of the outer retina between day and night due to the retinomotor movements, previously described in several species, including zebrafish [18, 19]. Interestingly, both the intensity and the location of the staining observed for the four studied genes were substantially different in retinae from animals sacrificed in the afternoon (17:00, Fig. 5a-d´´) versus the middle of the night (4:00, Fig. 5e-h´´). Due to this, the photoreceptor naming will be done only in the day retinae (Fig. 5a-d´´). ISH experiments revealed staining only in photoreceptor cells, throughout the entire retina. Expression of *pde6ga* (Fig. 5a-a´´) and *pde6gb* (Fig. 5b-b´´) were observed in the myoid and cytoplasm surrounding the nuclei of rods. Staining in the SSC seems to be present in A´-A´´ and B´-B´´ and we cannot discard that *pde6ga* and *pde6gb* are expressed in rods and also SSC. However, if true, this will not be in the entire retina (see Fig. 6). Expression of *pde6ha* (Fig. 5c-c´´) and *pde6hb* (Fig. 5d-d´´) was observed in all cones. The yellowish colour observed in **5g´´** could suggest double staining; however its absence in the day retina, combined with the around 100 fold lower expression levels (see next section) and the loose morphology of the night retinae led us to conclude that this is just an artefact. Additionally, double ISH experiments revealed coexpression of *pde6ga* and *pde6gb* in rods (Fig. 6a-c) and *pde6ha* and *pde6hb* in cones (Fig. 6d-f), throughout the retina. Expression of the four genes was also observed in the pineal complex (Fig. 5i-l) without noticeable differences between day and night (data not shown). However, to reveal any differential expression in the pineal complex, qRT-PCR must be performed.

In addition to the analyses on adults, ontogenetic analyses using 1–6 dpf larvae were performed by whole-mount ISH (WISH). The results reveal a synchronised onset of expression in the pineal complex at 1 dpf for all genes, except *pde6ha* that starts at 2 dpf, and delayed onset of expression for all of them until around 2–3 dpf in the retina (Additional file 3: Figure S18). The expression in the retina starts in the ventral part, in accordance with the differentiation process that starts in this region in zebrafish, different to most vertebrates, in which it is initiated in central locations [20].

### The zebrafish PDE6 inhibitory subunit paralogs show differential oscillatory patterns in expression levels for both rods and cones in adult retinae

qRT-PCR experiments were performed to analyse the expression levels of all zebrafish PDE6 genes in adult eyes from animals collected at six different time points during 24 h. The results were normalised to the mean of the housekeeping genes (*actb1* and *tuba1b*), previously reported to be appropriate for gene expression analysis in zebrafish [21]. For detailed *p-*values see Additional file 3: Table S3. Amplification of *pde6i* gene started between Cq 28 and 34, when using ten times higher amount of adult eye cDNA template (100 ng) for the 5 p.m. time point, indicating low expression levels. *Pde6i* expression levels were also investigated in 3dpf, 6dpf and 10dpf embryos, showing amplification between Cq 29 and 34, either using 10 ng or 50 ng of cDNA per reaction. Additionally, when searching zebrafish EST sequences using tblastn with the mRNA sequence for *pde6i* (GenBank accession no. XM_003198119.3), no identical sequences were found. Therefore, *pde6i* was excluded from expression pattern analyses.

**Fig. 7:**
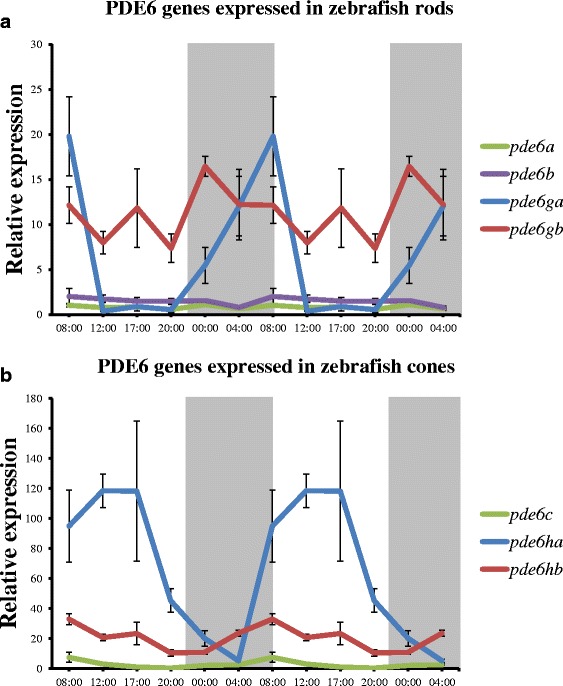
Day-night variation in relative expression levels of PDE6 genes in zebrafish. Double plots of mean relative expression over 24 h for rod- (**a**) and cone-specific (**b**) PDE6 genes. **a** The levels of *pde6a* and *pde6b* do not vary, while for *pde6ga* there is a drastic difference between day and night, being its highest expression levels in the early morning (8:00), after a constant expression increase during the night. Small variation was observed for *pde6gb* levels, being its highest levels at early night (00:00). **b** The expression levels of *pde6c* do not vary, while there is a ~20 fold variation for *pde6hb* between day and night, being its expression peak in the early morning (8:00) with a gradual reduction during the day. The variation for *pde6ha* is more drastic; there is a peak of expression in the middle of the day (17:00) and a subsequent ~120 fold reduction until the middle of the night (4:00). Expression levels for each gene are relative to the mean of zebrafish *actb1* and *tuba1b* (2^-ΔCq^) and error bars represent standard deviations. Shaded areas represent the dark period of a 24-h cycle. Note that both graphs are at different scales in order to be able to visualise the high expression levels of *pde6ha* during the day

In rods (Fig. 7a), significant (*p* < 0.05) but minor oscillations in gene expression (~2 fold) were observed over 24 h for the 2R duplicates *pde6a* and *pde6b*, which code for the rod-specific catalytic subunits. Interestingly, the 3R paralogs *pde6ga* and *pde6gb*, which code for the inhibitory subunits, behave quite differently: *pde6ga* has the highest expression in the early morning (08:00, *p* < 0.05), after a constant ~20 fold increase since late evening (20:00). Its expression decreases rapidly in the morning (08:00–12:00), to levels comparable to *pde6a* (Fig. 7a). The expression levels of *pde6gb* also oscillate, but differently in time and levels: its highest expression is early at night (24:00), after a quick ~10 fold increase since late evening (20:00, *p* < 0.05). After 24:00, its expression diminishes until noon (12:00). During the day, there is a fairly constant expression for both paralogs; *pde6gb* being ~10 fold higher than *pde6ga*.

In cones (Fig. 7b) the *pde6c* gene, which codes for the catalytic subunit, shows a minor oscillation in its expression during the day (~5 fold), with a significant peak of expression in the early morning (08:00) (Fig. 7b, *p* < 0.05). However, this oscillation is minimal compared to the oscillation in the expression *pde6ha*, coding for an inhibitory subunit, which gradually increases its expression (~120 fold) from the middle of the night (04:00) to reaching its peak in the evening (17:00, *p* < 0.05), to decrease again after 17:00. An oscillation of ~20 fold in the expression of *pde6hb* was also observed, increasing expression during the night (24:00–08:00) and decreasing during the day (08:00–20:00) (Fig. 7b).

## Discussion

**Fig. 8:**
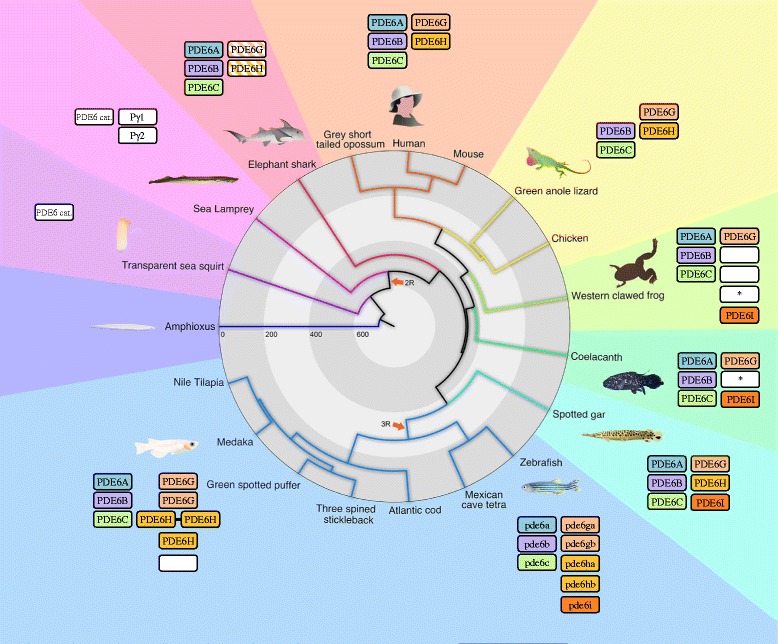
Phylogenetic tree summarizing the evolution of the PDE6 genes in vertebrates. The figure illustrates the expansion of the PDE6 catalytic (left columns) and inhibitory (right columns) gene families during vertebrate evolution and shows the approximate divergence times of species included in the analyses. No representative for any of these gene families were found in Amphioxus. The ancestral PDE6 catalytic subunit gene appeared in the common ancestor of tunicates (represented by a sea squirt) and vertebrates. On the contrary, the ancestral PDE6 inhibitory subunit gene appeared in the common ancestor of all vertebrates, after the split from tunicates. Both gene families expanded in 2R and were subsequently subjected to losses resulting in triplets for the catalytic (*PDE6A*, *PDE6B* and *PDE6C*) and the inhibitory subunit genes (*PDE6G*, *PDE6H* and *PDE6I*). In the sea lamprey, additional losses occurred resulting in one catalytic subunit gene and two inhibitory subunit genes. Similarly, one inhibitory subunit gene (*PDE6I*) was lost in cartilaginous fish (represented by the Elephant shark) and amniotes (represented by the grey short-tailed opossum, human, mouse, green anole lizard and chicken). In non-amniote lobe-finned fish (represented by the western clawed frog and the coelacanth) and ray-finned fish (represented by the spotted gar and all teleosts), all three PDE6 catalytic and three PDE6 inhibitory subunit genes are preserved. Teleost fish retained extra duplicates for some of the inhibitory subunit genes after 3R, as well as from local duplications. Finally, *PDE6A* was lost in the lineage leading to birds and non-avian reptiles. For consistency purposes, the colour code for the gene boxes is the same as used in Figs. 1, 2, Additional file 3: Figures S1-S17. Empty white boxes means that the orthology assignment could not be done confidently and boxes with an asterisk indicates fragmented sequences

This report describes the evolutionary history of all PDE6 genes as deduced from analyses of a wide range of vertebrate genomes. A résumé of their evolution, including the number and type of genes present in each vertebrate group, is shown in Fig. 8. In addition, zebrafish was used as a model to investigate the possible sub- or neofunctionalisations of PDE6 inhibitory subunit gene 3R paralogs.

### The PDE6 catalytic subunit gene repertoire is well conserved within the subphylum Vertebrata

The phylogenetic analysis of the PDE6 catalytic subunit gene family and our data on conserved synteny (including phylogenies for the neighbouring gene families) support an expansion in 2R for this gene family (Figs. 1 and 2). The three-gene repertoire (*PDE6A*, *PDE6B* and *PDE6C*) is well conserved across vertebrates, with the exception of northern hemisphere lampreys, that have only one gene and reptiles and birds that lack *PDE6A*. Additionally, the teleost-specific genome doubling did not result in any extra duplicates in this gene family, with the possible exception of the pufferfishes, which have an additional *PDE6C*.

Within the phototransduction cascade, our group has previously reported similar cases of gene repertoire conservation for the visual opsin gene family [22] and the alpha *transducin* gene family [23]. Altogether, these results reflect the high degree of conservation of the catalytic function, with no or little selection pressure to retain 3R duplicates; there might even have been selection against their retention.

Functionally, the three PDE6 catalytic subunits have similar catalytic activity and interaction with the PDE6 inhibitory subunits [24]. The difference is the efficiency of activation by transducin, in that rod PDE6 is activated much less efficiently than cone PDE6 [24]. The lack of a *PDE6A* gene in birds and non-avian reptiles could be explained by a loss in their common ancestor before the divergence of these lineages. Such a gene loss would be in line with a previous study of the chicken retina [25] which suggests that chicken uses a PDE6ββ homodimer instead of the PDE6αβ heterodimer commonly used by other vertebrates [2, 25].

### The appearance of an inhibitory subunit that regulates the PDE6 activity facilitated the success of the vertebrate visual system

The absence of PDE6 inhibitory subunit genes in invertebrates and their location in the *SSTR2*, *3, 5* and *UTSR* paralogon [16, 17], has led us to propose the following scenario for the evolution of this gene family. An ancestral PDE6 inhibitory subunit gene emerged *de novo* in the vertebrate lineage closely before 1R facilitating the shutting off of the PDE6 enzyme during dark conditions [2]. The appearance of *de novo* genes from non-coding genomic regions has been suggested to be important mechanism contributing to the origin of genes, where they can evolve important and even essential functions rapidly [26]. Subsequently, this gene was quadrupled in 2R followed by a loss of one of the four resulting genes.

Within gnathostomes, we here show for the first time that actinopterygians and non-amniote sarcopterygians have retained a third gene, that we named *PDE6I* to mark its paralogous relationship with *PDE6G* and *PDE6H*. We also found extra duplicates of *PDE6G* and *PDE6H* in teleosts, both resulting from 3R and through local duplications. In zebrafish we found that both the *pde6g* and *pde6h* genes have retained 3R duplicates. We have named the genes *pde6ga*, *pde6gb*, *pde6ha* and *pde6hb*. Additionally, zebrafish has the *pde6i* gene for which we detected very low expression in adult eyes and whole embryos up to 10 dpf, therefore we suggest that it might not have a function or, in adults, it is expressed in another organ.

The different intron losses that occurred in the *PDE6H* genes within teleosts can be the result of incomplete retrotranscription of the mRNA or retrotranscription of a partially spliced pre-mRNA, followed by replacement of the gene resulting in loss of one or more introns [27]. Similar processes has been observed in many genes [28–31]. It has been observed that the hatching enzyme genes with the highest expression levels are more prone to intron loss [29]. This is in line with our analyses that the expression of the *pde6ha* gene is much higher than the other genes (Fig. 7).

Among non-amniote sarcopterygians, we found *PDE6I* in the Western clawed frog and the coelacanth. In coelacanth *PDE6I* has probably been pseudogenised due to a frame-shift mutation that disrupts normal translation. However, due to the high overall sequence conservation of the coelacanth gene, the mutation is either recent or due to a sequencing error.

The sea lamprey genome assembly (see Table 2) has low coverage (5X) and is based on somatic cell DNA. This poses a problem because it is known that the lamprey genome goes through programmed loss of large amounts (~20 %) of somatic DNA in different cell lineages during embryonic development [32]. Thus, genes might be present in the genome but missing in the genome assembly. When searching the germline genome assembly of the arctic lamprey we found fragmented PDE6 inhibitory subunit gene sequences. The lifestyle of the northern hemisphere lampreys, mostly in pelagic and benthic waters [33], probably has led to losses in genes related to vision. Thus, to fully understand the visual gene repertoire, a genome assembly from a southern hemisphere lamprey, such as *Geotria australis*, living predominantly in surface waters [33] and known to have retained all ancestral subtypes of visual opsins [22, 34, 35], is needed.

### What did the visual system of zebrafish gain by retaining PDE6 inhibitory subunit duplicates after 3R?

The retention of duplicated genes may have two possible outcomes; subfunctionalisation, whereby the functions and/or expression of the ancestral gene are partitioned between the duplicates, or neofunctionalisation, whereby one or both of the copies gain novel functions [36, 37].

Here we demonstrate that the paralogs retained after 3R in zebrafish for the PDE6 inhibitory subunits, *pde6ga, pde6gb, pde6ha* and *pde6hb*, did not undergo subfunctionalisation involving topographical or temporal specialisations during development, i.e. the four genes are expressed in the retina and the pineal complex from early development. Instead, both paralogous pairs are coexpressed in the same retinal photoreceptor cell types as their amniote orthologs; *pde6ga* and *pde6gb* in rods and *pde6ha* and *pde6hb* in cones. An exception could be the SSC (UV-opsin containing cones) which possess some rod-like physiology features (see [38]) that could explain the possible expression of the four PDE6 inhibitory subunits, in addition to the previously suggested *gnb1a* and *gnb1b* [39] and *rcv1a* [40]. Further analyses are planned to be conducted in this sense in order to solve the transcriptome of each zebrafish cone type.

We also observed a striking difference in expression levels between day and night. Altogether, the high sequence identity, coexpression and different expression levels of the paralogs led us to consider neofunctionalisation as unlikely to have happened. Instead we suggest that the overall regulatory function by the PDE6 inhibitory subunits of the phototransduction cascade is most likely maintained, while specialisation of one or several of mechanisms of this regulation may be affected [12], with a direct effect on gene expression levels.

Similar coexpression in rods of paralogs involved in the phototransduction cascade has also been found for the *gnb1a* and *gnb1b transducin* subunit genes [39] and has been suggested for the *arrSa* and *arrSb* arrestin genes [41] and the *grk1a* and *grk1b* opsin GPCR kinase genes [42]. Additionally, subfunctionalisation of the zebrafish cone-specific arrestins has been reported previously: *arr3a* expressed in double cones and *arr3b* in single cones [41]. The current study shows a case of retention of two 3R paralogs in all cones similarly to the recently reported *recoverin* genes [40] which also might have been duplicated in 3R.

Altogether, zebrafish has retained several 3R duplicates of phototransduction cascade components that are coexpressed in the same photoreceptor cell types, indicating a dosage effect in regulation of the visual function. In this article, we show that the coexpression of PDE6 inhibitory subunit genes, inhibiting the main effector of the phototransduction cascade, is subfunctionalised by showing a daily oscillation in gene expression (see next section).

The pineal complex of non-mammalian vertebrates has rod- and cone-like photoreceptor cells [43, 44]. The expression of the four PDE6 inhibitory subunit genes in the pineal complex agrees with previous observations of the presence of several other components of the phototransduction cascade [39, 40, 45–47]. Altogether, their expression further supports the idea that the pinealocytes share a common origin with the retinal photoreceptors [38, 39, 48]. However, the functional role for these duplicated genes remains unclear.

The onset of expression for the PDE6 inhibitory subunit genes is earlier in the pineal complex than in the retina, similar to other components of the teleost phototransduction cascade like opsins [49–51], transducins [39] or cyclic nucleotide gated channels (CNGs) (unpublished results) and recoverins [40]. This is in accordance with the involvement of the pineal complex in regulation of hatching in Atlantic halibut [51]. The delay of expression in the retina is consistent with previous reports that have shown the zebrafish retina to be responsive to light stimuli around 3 dpf [52].

### Opposite rhythmic oscillations in expression for the PDE6 inhibitory subunit genes are related to differential light sensitivity

Circadian changes in the zebrafish and cichlid retinae have been previously reported regarding morphology [18, 19], gene expression [53, 54] and physiology [54, 55]. How those circadian changes influence visual function is not well understood. Here we describe gene expression data of regulatory genes that are likely to directly influence the sensitivity to light.

**Fig. 9:**
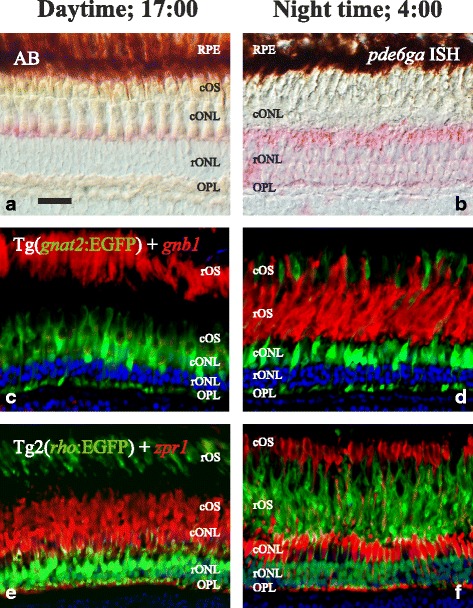
Retinomotor movements in the zebrafish retina. Photomicrographs of adult zebrafish retinae showing morphological differences between day (17:00; left column) and night (4:00; right column). In the upper row, bight field photomicrographs taken using Nomarski contrast show the clear stratification during daytime (**a**), different to the poorly stratified retina at night (**b**). In both pictures, Fast Red staining, in pink, shows the presence of *pde6ga* mRNAs in rods. Note the different intensity in staining, confirmed by qRT-PCR as higher expression at 4:00 AM (Fig. 7). **c**-**f** pictures are fluorescence photomicrographs of immunostainings illustrating the retinomotor movements with photoreceptor-specific markers. In **c** and **d**, sections of a transgenic line that expresses EGFP in cones: Tg(*gnat2*:EGFP) were incubated with a rod-specific anti-GNB1 antibody (in red). In **e** and **f**, sections of a transgenic line that expresses EGFP in rods: Tg2(*rho*:EGFP) were incubated with a double cone-specific anti-*zpr1* antibody (in red). In both cases, note the position of the rod outer segments in the outermost part of the retina at daytime, while at night they have moved to inner positions and the cone outer segments have moved outwards. DAPI was used as a nuclei counterstain (**c**-**f**). RPE: retinal pigment epithelium. For more abbreviations, see Fig. 5 legend. Scale bar is 20 μm

Zebrafish have been reported to show circadian oscillations in visual sensitivity [55], which can be partially explained by the striking morphological differences between day and night in the outer retina [19]; rod outer segments (rOS) are located in outer locations during the day and move inwards at night, while cone outer segments (cOS) are located in the innermost outer retina during the day and move outwards at night (see Fig. 9). These retinomotor movements, present in most vertebrate groups except mammals [56], facilitate an optimal light exposure for each photoreceptor type at the different light conditions, which increases their sensitivity, albeit losing resolution at night [19].

Putting together data about phototransduction cascade proteins, cone opsins [54] and *pde6ha* (*present results*) have their highest expression in the late afternoon, when the cones mediating photopic vision have high sensitivity [55]. The cone transducin levels, on the contrary, do not oscillate significantly (unpublished results). High levels of opsins would increase sensitivity while *pde6ha* would be a key factor for the non-saturating effect, exclusive for cones [38], tentatively by the binding of the excess of free PDE6 inhibitory subunits to activated alpha transducins in a desensitisation-like effect. For scotopic vision, rhodopsin (unpublished results) and rod transducin [39] expression levels do not oscillate significantly, while expression of both *PDE6G* paralogs is higher in rods at night, where dim-light conditions activate rods.

Oscillations in the expression of opsins have been reported in teleosts [53, 54], frog [57] and chicken [58], mice [59], the Sudanian grass rat [60] with no obvious common denominator among all these results. These circadian changes in gene expression have been attributed to the circadian cycle of outer segment disc shedding of the photoreceptors [53] or correlated with behavioural sensitivity to light [54]. This is the first report showing gene expression oscillations in a phototransduction cascade protein except opsins. Similarly, oscillations in gene expression has been observed for the interphotoreceptor retinoid binding protein, a gene specific for photoreceptors but not strictly phototransduction cascade related protein, in both zebrafish [61] and chicken [62].

In this study we investigated the mRNA expression pattern and expression levels due to the difficulty to generate specific antibodies able to target the 3R paralogs. Therefore, we have to assume that there is no or little delay in the translation of proteins or in the trafficking to and throughout the outer segment. Contrarily, it has been previously reported that transducin is stored in the photoreceptor inner segments in rats before being transported to the outer segments during the night [63]. However, this has not been demonstrated in other species or for other proteins of the phototransduction cascade [39]. In order to investigate the light-dependence of the PDE6 inhibitory subunits oscillations in gene expression levels and its effect on visual sensitivity, our group has initiated functional and semi-functional studies.

## Conclusions

We report here that both catalytic and inhibitory subunit gene families of PDE6 were duplicated in 2R, resulting in cone- and rod-specific copies. Together with duplications of several other gene families expressed in the eye [3, 4, 22, 23, 41] this emphasises further the importance of 2R for vertebrate evolution in general and particularly for vision. The 2R event facilitated the differentiation of rods and cones by subfunctionalisation and/or neofunctionalisation of gene duplicates. Additionally, we report that the important regulatory PDE6 inhibitory subunit genes seem to have arisen in conjunction with the origin of the vertebrates, shortly before the genome quadruplication in 2R, presumably facilitating the rapid regulation of visual responses of the vertebrate eye mediated by the PDE6 enzyme. After 2R, the inhibitory subunit gene duplicates became specialised on rods and cones, respectively, and a third ancient copy presumably acquired distinct functions and still exists in some vertebrate lineages, although not amniotes. Finally, we show that the 3R-generated PDE6 inhibitory subunit gene duplicates evolved dramatically different daily expression profiles in zebrafish. Thus, the PDE6 inhibitory gene duplicates display novel specialisations that warrant further functional experiments.

## Methods

### Collection of amino acid sequences for phylogenetic analyses

Amino acid sequence predictions of the PDE6 subunit genes were downloaded from the Ensembl genome browser version 69 (October 2012) and the Pre! Ensembl genome browser from the species listed in Table 2. In addition human PDE6 subunit amino acid sequences were used as TBLASTN queries for searches against the genome assemblies of the species where genes were missing. Regions in the genome assemblies with BLAST hits, but without any Ensembl gene prediction, were downloaded and predicted using GENSCAN [64] (available at the web server: http://genes.mit.edu/GENSCAN.html) or predicted manually following splice donor-acceptor sites and sequence homology. The sequences for the human *PDE5A* and *PDE11A* genes were included in the analysis to serve as outgroup to root the trees.

In order to identify invertebrate orthologs of the PDE6 inhibitory subunit genes, human *PDE6G* and *PDE6H* amino acid sequences were used as queries in TBLASTN searches against the whole genome shotgun (WGS) sequences database from the National Centre for Biotechnology Information (NCBI) where all vertebrate sequences was excluded to try to identify invertebrate homologs. Hidden Markov Model (HMM) searches were performed against protein and nucleotide databases both on http://hmmer.janelia.org and locally [65]. A HMM based on a nucleotide alignment of human, chicken and sea lamprey PDE6 inhibitory subunit gene sequences was generated using a local installation of *hmmbuild* and used as input to a local installation of *nhmmer* against several invertebrate genomes listed in Table 2.

### Alignments and phylogenetic analyses

Amino acid sequences of each protein family were aligned using ClustalO with standard settings within the Seaview 4.5.3 program [66]. Stretches of poorly aligned sequences, that seemed to be the result bad sequence predictions, were checked towards the genomic sequence. To identify the correct prediction of these parts genscan was used for the region of the gene. If genscan did not find the sequence, manual annotation was tried following sequence homology as well as consensus donor and acceptor splice sites in the genomic sequence in combination with TBLASTN searches.

Alignments used for phylogenetic analysis were tested for the best amino acid substitution model using ProtTest 3.2 [67] with the following settings; Starting topology: Fixed BioNJ JTT, Matrices: JTT, LG, DCMut, Dayhoff, WAG, RtREV, CpREV, Blosum62 and VT.

PhyML trees were created using the PhyML 3.0 web server [68] (available at: http://www.atgc-montpellier.fr/phyml/) or locally using Seaview 4.5.3 using the following settings; JTT was used as substitution model for all of the neighbouring gene families while WAG was used for the PDE6 catalytic subunit gene family. Equilibrium frequencies were set to empirical, proportion of invariable sites estimated, number of substitution rate categories eight, gamma shape parameter estimated, starting tree BIONJ, type of tree improvement SPR & NNI, number of random starting trees no, optimise topology yes, optimise branch lengths yes, compute aLRT no and finally bootstrap was used with 100 replicates.

### Analysis of conserved synteny

To analyse the conservation of synteny for the chromosomal regions harbouring the PDE6 catalytic subunit genes, information on associated gene names, Ensembl gene IDs, Ensembl transcript IDs, genomic location and Ensembl protein family IDs were downloaded from Ensembl version 61 in a region spanning approximately 10 Mb upstream and downstream of the *PDE6A*, *PDE6B* and *PDE6C* genes in the human genome. The chosen region for human chromosome 4 was 1 bp to 10.7 Mb (*PDE6B*), 139.2 Mb to 159.3 Mb for chromosome 5 (*PDE6A*) and 85.4 Mb to 105.4 Mb for chromosome 10 (*PDE6C*). Ensembl protein families with members in at least two of the selected chromosomal regions were selected for subsequent phylogenetic analysis and sequences were retrieved from Ensembl version 65.

The PDE6 inhibitory subunit genes were upon initial inspections in tetrapod genomes found to be located in the *SSTR2, 3* and *5* paralogon previously described by [16]. Thus, we were able to use their supplementary chromosomal tables to assign the orthology of most of the PDE6 inhibitory subunit genes. To investigate the conservation of the paralogon in the spotted gar genome, a species not included in their analysis, we investigated a selection of neighbouring gene families in regions spanning 5 mega base pairs (mb) upstream and downstream of the spotted gar PDE6 inhibitory subunit genes. We identified and analysed several of the neighbouring gene families they described, indicating that the PDE6 inhibitory subunit genes indeed are located in the same paralogon in the spotted gar (data not shown). Additionally, to be able to assign the orthology of the PDE6 inhibitory subunit genes located in difficult regions (too low resolution in the [16] analysis) and on short scaffolds, lists of spotted gar genes, also containing Ensembl’s predicted orthologs and their locations in other species, located in regions 1 Mb upstream and downstream of the spotted gar PDE6 inhibitory subunit genes were downloaded from Ensembl version 75. The included species in this orthology analysis were the Western clawed frog, coelacanth, zebrafish, three-spined stickleback, Nile tilapia, Atlantic cod and medaka.

### Animals used for gene expression analyses

The animals used in this study were from three zebrafish lines: AB strain zebrafish (*n* = 45) purchased from the Science for Life Laboratory Zebrafish Technology Platform (Uppsala University, Sweden), Tg(*gnat2*:EGFP) zebrafish (*n* = 7) [69] Tg(*rho*:EGFP) (*n* = 8) [70]. All adult zebrafish used in the experiments were from 6 to 18 months old and were maintained under standard conditions with lights on at 8 a.m. and off at 10 p.m.in a 14–10 light–dark cycle and the change of lighting conditions was abrupt.

### Quantitative reverse-transcriptase PCR

For quantitative reverse-transcriptase PCR (qRT-PCR) experiments, the fish were anesthetised using Tricaine (0.04 % in system water) and decapitated. The heads were immersed in RNA*later®* (Qiagen: cat. no. 76104) prior to dissection of the eyes. Both eyes of each individual were pooled together for RNA extraction. Total RNA was extracted using the RNeasy mini kit (Qiagen: cat. no. 74104). Three to four individuals were collected at six time points during the day: 08:00, 12:00, 17:00, 20:00, 00:00, 04:00. Following RNA extraction, the RNA was precipitated in ethanol for increased purity, treated with DNase I (Thermo Scientific: cat. no. EN0521) and used as template for reverse transcription using iScript™ cDNA synthesis kit (Bio-Rad: cat. no. 170–8890) in both RT and no-RT reactions.

Each qRT-PCR reaction was set up as follows: 10 μl iQ™ SYBR® Green Supermix (Bio-Rad: cat. no. 170–8880), 1,25 μl forward- and 1,25 μl reverse primers (10 pmol/μl), 6,5 μl MilliQ H_2_O and finally 1 μl 10 ng/μl cDNA or MilliQ H_2_O. Each reaction was run in triplicates, together with no-RT and no-template controls in duplicates. Primers were designed using primer-blast (http://blast.ncbi.nlm.nih.gov) spanning either exon-exon boundaries or placed in separate exons (Table 3). Actin beta 1 (*actb1*) and alpha tubulin 1b (*tuba1b*) were used as housekeeping genes according to [21] with our own primer pairs. qRT-PCR efficiency and amplification data was analysed using LinRegPCR version 2014.6 [71] and presented as relative to the mean of *actb1* and *tuba1b* (the 2^-ΔCq^ method). Statistics were analysed for each gene using one-way ANOVA with a Tukey’s Multiple Comparison post-hoc test in GraphPad Prism 5.00. The mean relative expression for each gene and time-point were plotted in double plots (Fig. 7).

**Table 3:** List of the primer pairs used for qRT-PCR experiments and to synthesise riboprobes

qRT-PCR primers			
Gene	Forward primer 5′–3′	Reverse Primer 5′–3′	Product length
*pde6a*	CAGTCAACAAGATCGGGGCT	GCTCAGGTGAAACACTCGGA	104 bp
*pde6b*	ACTCACGACAGGCAAACTGA	CATGCAGCTTGGCTAGAGGA	146 bp
*pde6c*	ACTCCTGATGGCAGGGAGAT	AGCAACATAGGTGGGCAGTC	135 bp
*pde6ga*	CACAAGGGCCCACCTAAGTT	AACTCCAGGTGACTGTACGC	164 bp
*pde6gb*	GTTCAAGAGCAAGCCCCCAA	GTGCCTAAACCTTCCATGCC	75 bp
*pde6ha*	CTTCGGAGACGACATCCCAG	ATCGCTGAGCTCCATGTCTC	94 bp
*pde6hb*	CCTGGACAGAAAGGGTTTGGT	CTGAGCTCCATGTCCCCGAA	104 bp
*pde6i*	ACAACTACACCCAGAAGCGG	TGCCAAGACCATCCATTCCT	127 bp
*actb1*	GGCACGAGAGATCTTCACTCCCC	CCATGCCAACCATCACTCCCTGA	195 bp
*tuba1b*	CGGAGCTGGAAAACACGTCCCC	TGGTCAGACAGTTTGCGAACCCTA	216 bp
Probe primers			
*pde6ga*	TCCACCAGCAACATCCTGCACC	CGCGCTATGGCAGACGCTGA	679 bp
*pde6gb*	TCTGCCATGTCCTCCATCGGC	AGACGAGGCACCGAGGCACA	381 bp
*pde6ha*	GGCGCTCTCAGGCCAACACA	AGGCACAAACACAATCTCATGCACA	205 bp
*pde6hb*	TGGCCAAATACGGCATCATCT	CATCCATCGTGGCTGCTACA	280 bp

The current table specifies the primer pair sequences used to amplify gene sequences either to perform qRT-PCR or to synthesise the antisense and sense riboprobes used in ISH experiments. Actin beta I (*actb1*) and tubulin alpha 1b (*tuba1b*) were used as housekeeping genes in the qRT-PCR experiments

For the ontogenetic analysis of *pde6i* expression levels, 3 pools of 20 embryos were isolated for 3dpf, 6dpf and 10dpf embryos. Subsequently, their mRNA was extracted, cDNA was synthesised, the RT-qPCR was performed and the results were analysed as is described above.

### Probe design and synthesis

In order to study the expression pattern of the four zebrafish PDE6 inhibitory subunit genes (*pde6ga, pde6gb, pde6ha and pde6hb*), antisense riboprobes were designed targeting their 3′ untranslated regions (3′UTR). PCR primers were designed using the Primer-BLAST tool [72], available at the NCBI webpage (http://blast.ncbi.nlm.nih.gov/Blast.cgi) (see Table 3) and PCR reactions were performed using genomic DNA from zebrafish. The resulting amplicons were cloned into pCR®II-TOPO® vectors (Invitrogen: cat. no. K4650-01), sequenced and used for probe synthesis with either T7 or SP6 RNA polymerase using DIG RNA labelling kit (Roche: cat. no. 11175025910) according to manufacturer’s instructions. Sense and antisense probes were synthesised and the former was used to control for specificity.

### In situ hybridisation (ISH)

The fish used in the ISH experiments were anesthetized using Tricaine (0.04 % in system water), either during the day (17:00) or during the night (04:00). The heads were dissected and fixed by immersion in 4 % paraformaldehyde (PFA) diluted in phosphate buffer 0.1 M pH 7.4 (PB) for 7 h and washed in phosphate buffered saline 0.1 M pH 7.4 (PBS) overnight, both at 4 °C. Subsequently the heads were cryoprotected in 30 % sucrose and sectioned in a cryostat (Microm Cryo-Star HM 560) obtaining transversal sections 12–20 μm thick stuck on positively charged slides.

The ISH was performed according to [73] with minor adaptations. The final staining reaction was carried out using different substrates for the AP enzyme bounded to the Fab fragments: NBT/BCIP or Fast Red tablets (Roche: cat. no. 11681451001 and 11496549001, respectively). Sense probes were incubated in parallel with the antisense, as a specificity control, with no staining as a result. In order to preserve the fluorescence of the EGFP when using transgenic animals, the hybridisation reaction time was reduced from overnight to 6 h. Double ISH experiments according to [74] were also performed in specific cases. Lastly, all slides were mounted using VectaShield® mounting medium with DAPI incorporated.

Cell assignment was based on overall cell morphology, topological location of the nuclei and the mitochondria dense ellipsoids [75] of the different photoreceptor cell types, as well as immunohistochemistry on rod- or cone-specific EGFP fluorescence transgenic lines. Rod-specific rabbit anti-GNB1 (Nordic BioSite: cat. no. LS-C90703; 1:500) and double cones-specific mouse anti-*zpr1* (ZIRC; 1:400) primary antibodies were used with donkey anti-rabbit coupled Alexa 555 (Life technologies cat. no. A31572; 1:1000) and donkey anti-mouse coupled Alexa 488 (Life Technologies cat. no. A21202; 1:1000) as secondary antibodies. We have used the nomenclature proposed by [76] for the different cone types: double cones (DC, middle and long wavelength), long single cones (LSC, short wavelength) and short single cones (SSC, ultraviolet).

Bright-field, fluorescence and Nomarski contrast photomicrographs, as well as their combinations were taken using a Zeiss Axioplan 2 microscope equipped with a Zeiss AxioCam camera or an inverted LSM510 Zeiss confocal microscope. The figures merged using CorelDRAW Graphics Suite X6.

### Whole mount in situ hybridisation (WISH)

All embryos and larvae (1–6 dpf) were collected as described previously [73]. The WISH experiments were performed in an InsituPro VSi (Intavis AG, Köln, Germany) ISH robot at the Science for Life Laboratory Zebrafish Technology Platform (Uppsala Universitet, Sweden). The staining was done outside of the robot using NBP/BCIP at 37 °C. The images were acquired using a stereomicroscope Nikon SMZ1500 with a Nikon DS-Vi1 camera and the figures merged using CorelDRAW Graphics Suite X6.

## Additional files

Additional file 1: Multiple sequence alignment in FASTA format for the amino acid sequences of the PDE6 catalytic subunit genes. This alignment was done using ClustalO in the Seaview 4.5.3 program and was one used for the phylogenetic analysis. Sequence names are provided in Additional file 2. (DOC 53 kb) Additional file 2: Tables containing sequence identifiers, genomic locations and sequence names. The tables include information for genes used in the phylogenetic analyses, both from the PDE6 subunit gene families and the identified neighbouring gene families. Each family has its own sheet in the excel file. (XLSX 58 kb) Additional file 3: Figures and Tables. This file lists all the figures (S1-S18) and tables (S1-S3). Figure S1: Phylogenetic maximum likelihood tree with additional PDE6 catalytic subunit gene amino acid sequences from bird and non-avian reptile genomes. No PDE6A genes could be identified in any of the investigated species, further supporting the loss of PDE6A in this lineage. Figure S2-S9: Phylogenetic maximum likelihood trees of the investigated neighbouring gene families of the PDE6 catalytic subunit genes. Figure S10-S17: Synteny comparisons between spotted gar linkage groups carrying PDE6 inhibitory subunit genes and chromosomes and scaffolds in other species where comparisons with tables from Ocampo Daza et al., 2012 were uninformative. Figure S18: Whole mount in situ hybridisation with PDE6 inhibitory subunit gene expression during zebrafish development (1dpf – 3dpf). Table S1: List of the identified neighbouring gene families of the PDE6 catalytic subunit gene paralogon. Table S2: Table listing the pair-wise percentage nucleotide sequence identity between the PDE6 inhibitory subunit gene 3ʹUTR sequences. Table S3: Table displaying the results from one-way ANOVA of the relative expression for each PDE6 subunit gene between the six time-points. (PDF 1990 kb) Additional file 4: Multiple sequence alignment in FASTA format for the amino acid sequences of the identified PDE6 inhibitory subunit genes. The alignment was done using ClustalO in the Seaview 4.5.3 program. Sequence names are provided in Additional file 2. (RTF 9 kb) Additional file 5: Synteny tables from Ocampo Daza et al. 2012 with our identified PDE6 inhibitory subunit genes added. Tables showing genomic locations for the PDE6 neighbouring gene families organized in chromosomal blocks from Ocampo Daza et al. 2012, to which we have added the genomic locations of the PDE6 inhibitory subunit genes we identified in the current study. (XLSX 87 kb)
