# Supplementary material for: Evolution and expression of the phosphodiesterase 6 genes unveils vertebrate novelty to control photosensitivity
Source: BMC Evol Biol. 2016 Jun 13;16:124. doi: 10.1186/s12862-016-0695-z (PMC4906994; doi:10.1186/s12862-016-0695-z)
Supplement: Additional file 3: Figures and Tables. — This file lists all the figures (S1-S18) and tables (S1-S3). Figure S1: Phylogenetic maximum likelihood tree with additional PDE6 catalytic subunit gene amino acid sequences from bird and non-avian reptile genomes. No PDE6A genes could be identified in any of the investigated species, further supporting the loss of PDE6A in this lineage. Figure S2-S9: Phylogenetic maximum likelihood trees of the investigated neighbouring gene families of the PDE6 catalytic subunit genes. Figure S10-S17: Synteny comparisons between spotted gar linkage groups carrying PDE6 inhibitory subunit genes and chromosomes and scaffolds in other species where comparisons with tables from Ocampo Daza et al., 2012 were uninformative. Figure S18: Whole mount in situ hybridisation with PDE6 inhibitory subunit gene expression during zebrafish development (1dpf – 3dpf). Table S1: List of the identified neighbouring gene families of the PDE6 catalytic subunit gene paralogon. Table S2: Table listing the pair-wise percentage nucleotide sequence identity between the PDE6 inhibitory subunit gene 3ʹUTR sequences. Table S3: Table displaying the results from one-way ANOVA of the relative expression for each PDE6 subunit gene between the six time-points. (PDF 1990 kb) [file 12862_2016_695_MOESM3_ESM.pdf]

EVOLUTION AND EXPRESSION OF PHOSPHODIESTERASE 6 GENES SHOWS VERTEBRATE NOVELTY IN THE CONTROL OF PHOTOSENSITIVITY

Supplementary figures and tables

In this file all the supplementary figures are listed (S1-S18) as well as all the supplementary tables (S1-S3).

- **Figure S1:** Phylogenetic maximum likelihood tree with additional PDE6 catalytic subunit gene amino acid sequences from bird and non-avian reptile genomes. No *PDE6A* genes could be identified in any of the investigated species, further supporting the loss of *PDE6A* in this lineage.
- **Figure S2-S9:** Phylogenetic maximum likelihood trees of the investigated neighbouring gene families of the PDE6 catalytic subunit genes.
- **Figure S10-S17:** Synteny comparisons between spotted gar linkage groups carrying PDE6 inhibitory subunit genes and chromosomes and scaffolds in other species where comparisons with tables from Ocampo Daza et al., 2012 were uninformative.
- **Figure S18:** Whole mount *in situ* hybridisation with PDE6 inhibitory subunit gene expression during zebrafish development (1dpf – 3dpf).
- **Table S1:** List of the identified neighbouring gene families of the PDE6 catalytic subunit gene paralogon.
- **Table S2:** Table listing the pair-wise percentage nucleotide sequence identity between the PDE6 inhibitory subunit gene 3′UTR sequences.
- **Table S3:** Table displaying the results from one-way ANOVA of the relative expression for each PDE6 subunit gene between the six time-points

S1 – PDE6 catalytic subunits family

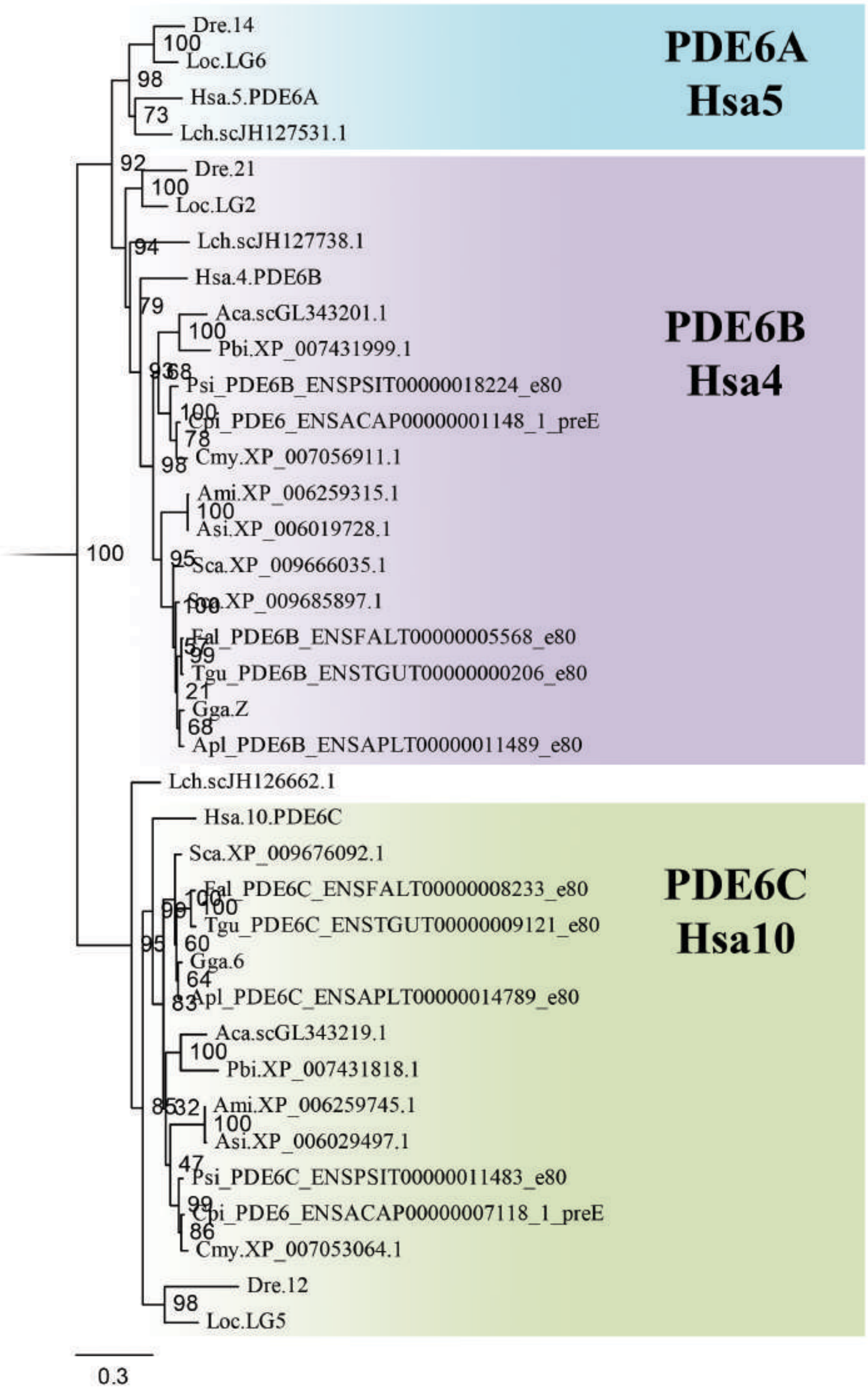

Supplementary figure S1: Phylogenetic maximum likelihood tree of the PDE6 protein family using 100 bootstrap and the WAG amino acid substitution model. The *PDE6B* and *PDE6C* but not *PDE6A* genes were identified in the investigated bird and non-avian reptile genomes. Species abbreviations are summarised in table 2, the number following the species abbreviation is either the chromosome or scaffold number or transcript id/accession number. The tree was rooted with the human PDE5A and PDE11A sequences (not shown).

S2 – STK32

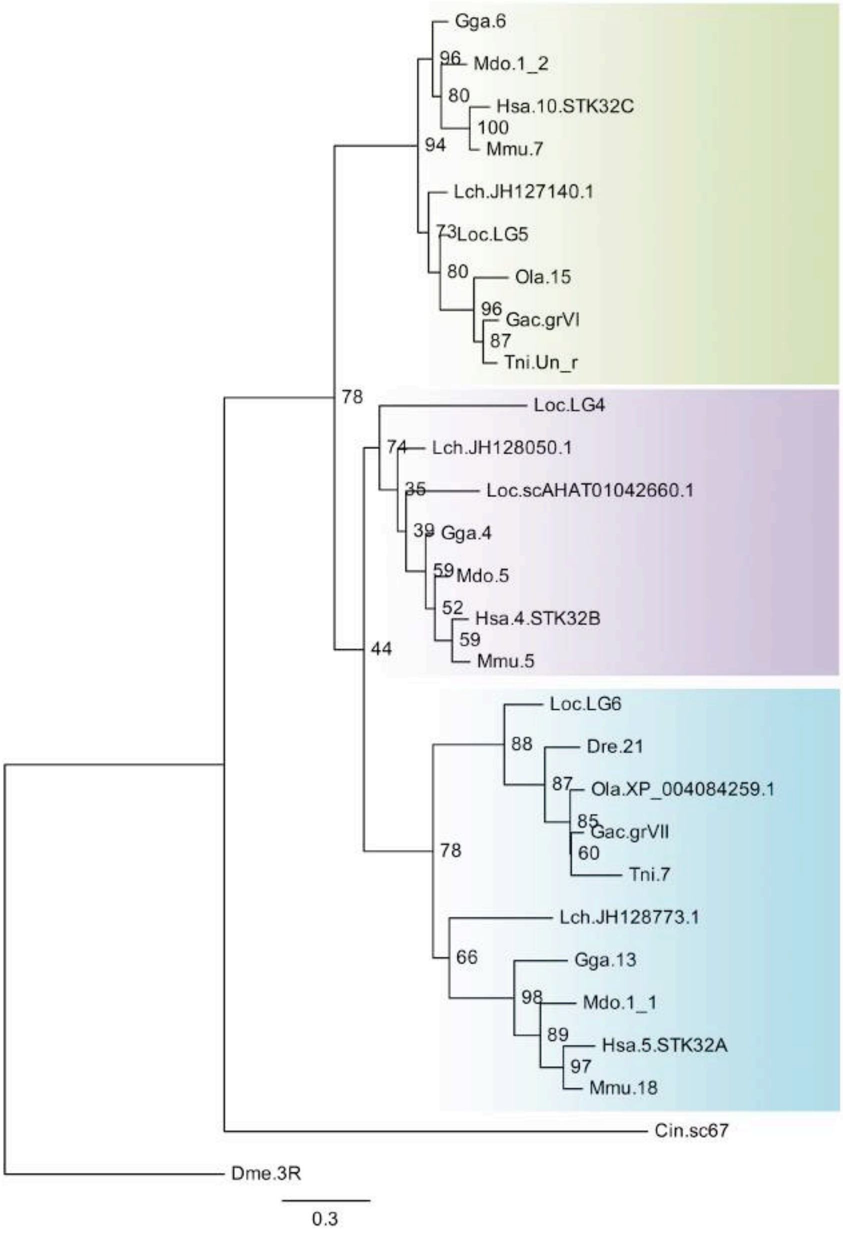

Supplementary figure S2: Phylogenetic maximum likelihood tree of the STK32 protein family using 100 bootstrap and the JTT amino acid substitution model. The tree was rooted with the fruitfly ortholog. The topology of the tree with three clusters is in line with an expansion in 2R. Teleost fish has lost the STK32B gene. Species abbreviations are summarised in table 2, the number following the species abbreviation is either the chromosome or scaffold number or transcript id/accession number.

S3 – TBC1D

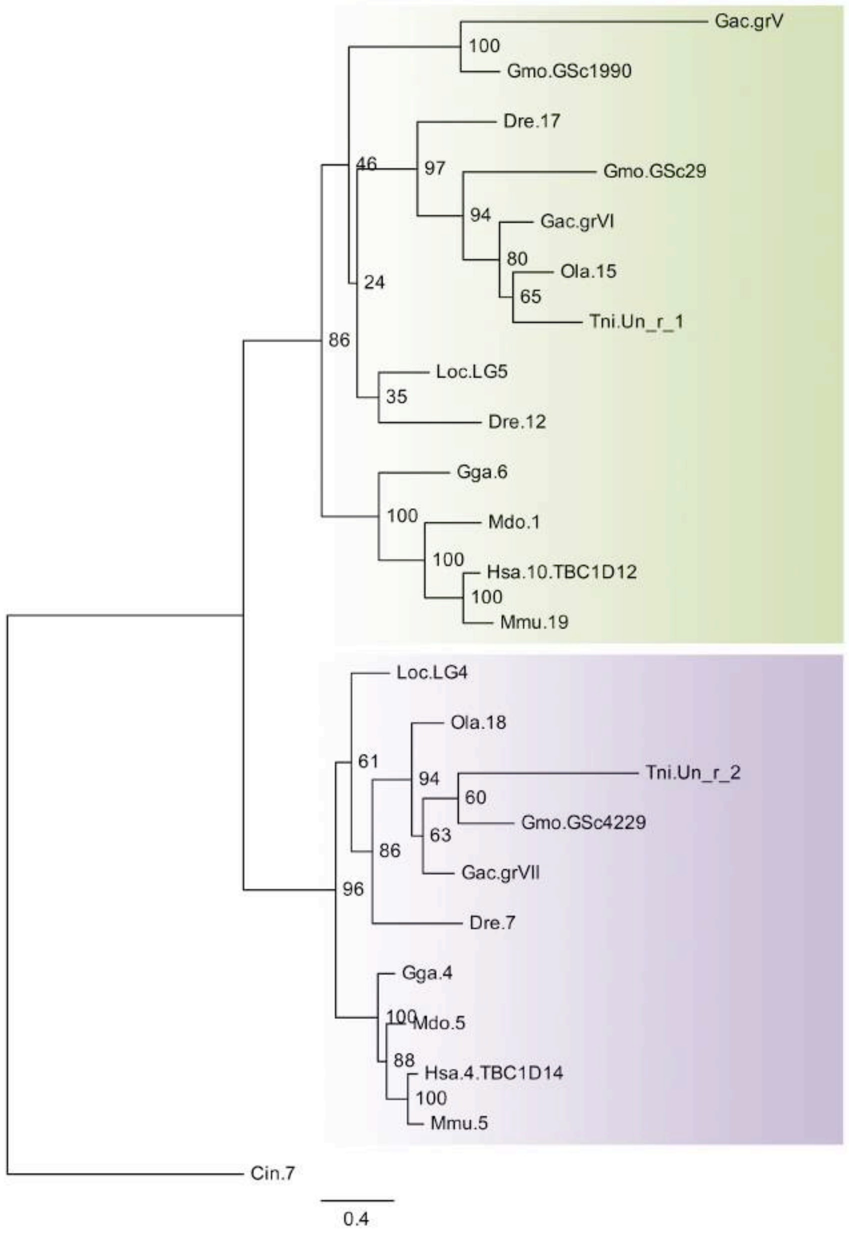

Supplementary figure S3: Phylogenetic maximum likelihood tree of the TBC1D protein family using 100 bootstrap and the JTT amino acid substitution model. The tree was rooted with the vase tunicate ortholog. The two vertebrate clusters in the tree are in line with an expansion in 2R, followed by losses. Species abbreviations are summarised in table 2, the number following the species abbreviation is either the chromosome or scaffold number or transcript id/accession number.

S4 – PPP2R2

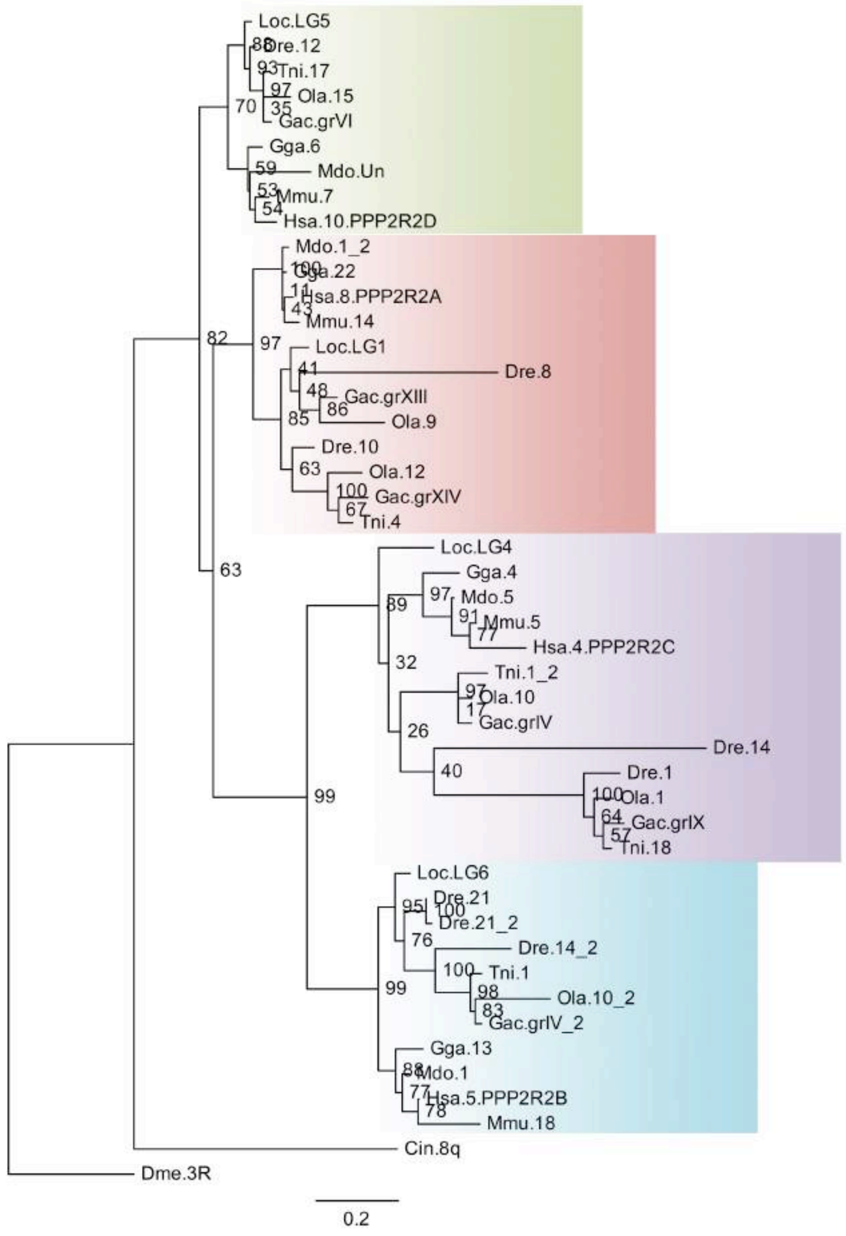

Supplementary figure S4: Phylogenetic maximum likelihood tree of the PPP2R2 protein family using 100 bootstrap and the JTT amino acid substitution model. The tree was rooted with the fruitfly ortholog. The topology, with four vertebrate clusters, is in line with an expansion in 2R. *PPP2R2A* and *PPP2R2C* probably duplicated further in 3R, although the support for *PPP2R2C* is weaker. Species abbreviations are summarised in table 2, the number following the species abbreviation is either the chromosome or scaffold number or transcript id/accession number.

S5 – DPYS

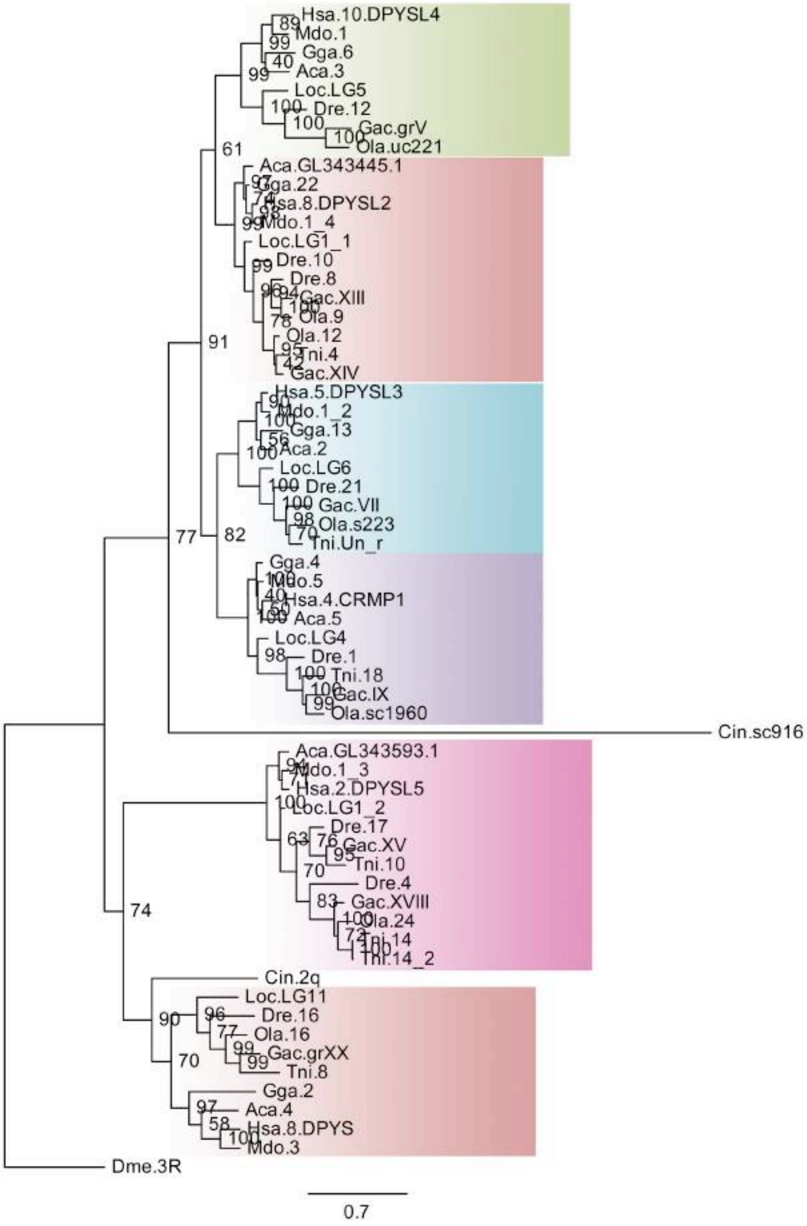

Supplementary figure S5: Phylogenetic maximum likelihood tree of the DPYS protein family using 100 bootstrap and the JTT amino acid substitution model. The tree was rooted with the fruitfly ortholog. The topology of the tree suggests a pre-vertebrate duplication followed by 2R expansion of one of the resulting genes. *DPYSL2* possibly duplicated further in 3R. Species abbreviations are summarised in table 2, the number following the species abbreviation is either the chromosome or scaffold number or transcript id/accession number.

S6 – ABLIM

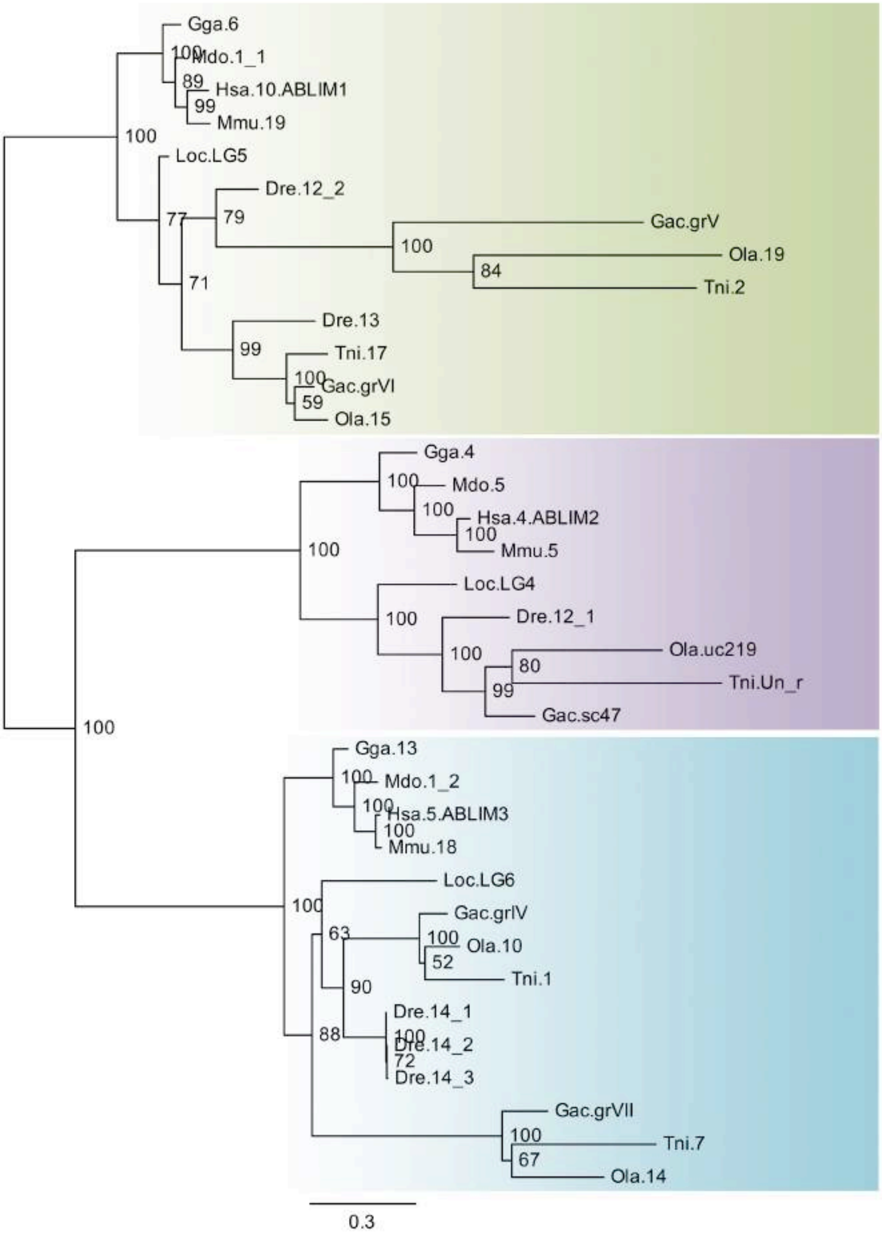

Supplementary figure S6: Phylogenetic maximum likelihood tree of the ABLIM protein family using 100 bootstrap and the JTT amino acid substitution model. The tree was midpoint-rooted. Due to the lack of invertebrate sequences for relative dating, it is not possible to say when the duplications that resulted in the three vertebrate clusters occurred. However, the presence of three clusters is possible if the family duplicated in 2R. *ABLIM1* and *ABLIM3* duplicated further in 3R. Species abbreviations are summarised in table 2, the number following the species abbreviation is either the chromosome or scaffold number or transcript id/accession number.

S7 – AFAP

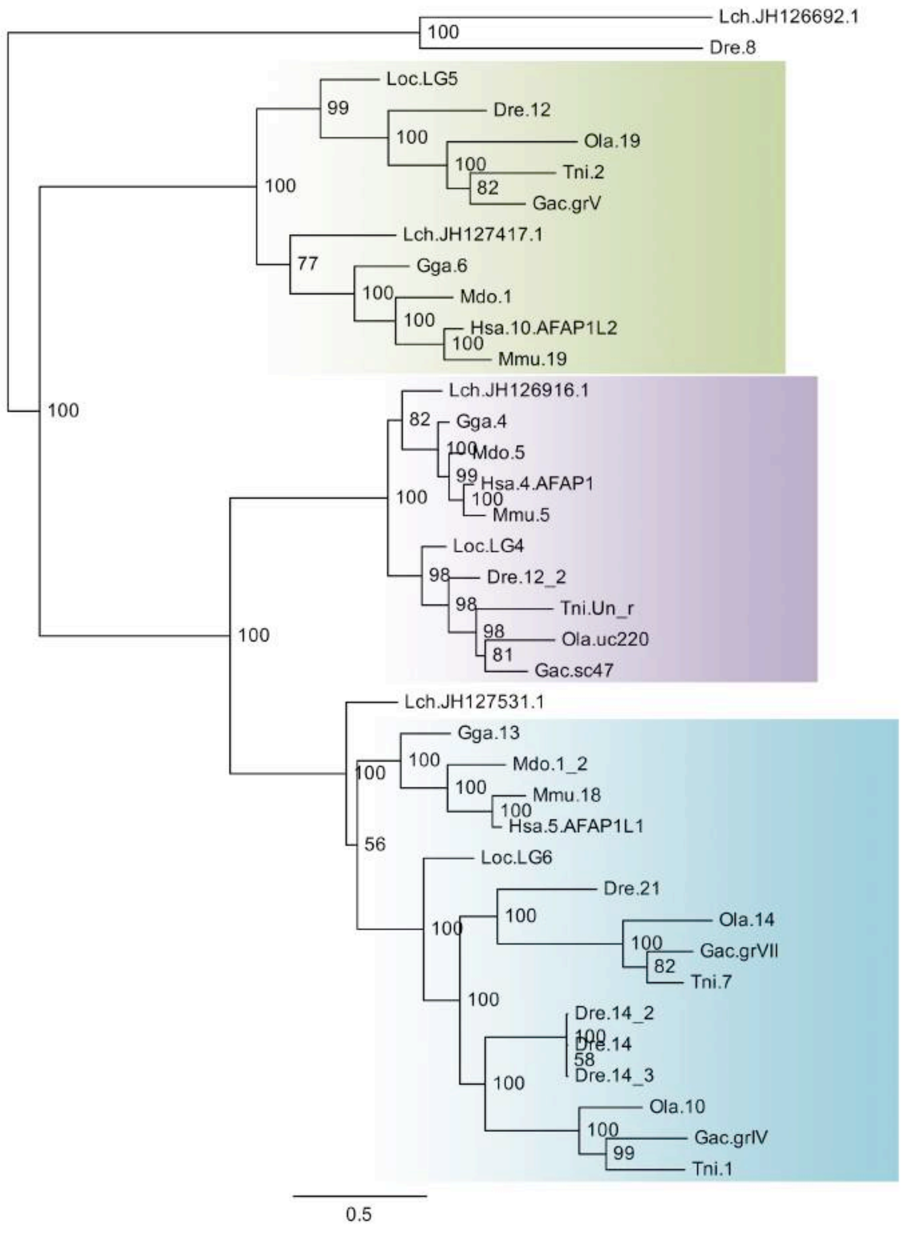

Supplementary figure S7: Phylogenetic maximum likelihood tree of the AFAP protein family using 100 bootstrap and the JTT amino acid substitution model. The tree was midpoint-rooted. Due to the lack of invertebrate sequences for relative dating, it is not possible to say when the duplications that resulted in the three vertebrate clusters occurred. However, the presence of three clusters is possible if the family duplicated in 2R. *AFAP1L1* duplicated further in 3R. Species abbreviations are summarised in table 2, the number following the species abbreviation is either the chromosome or scaffold number or transcript id/accession number.

S8 – JAKMIP

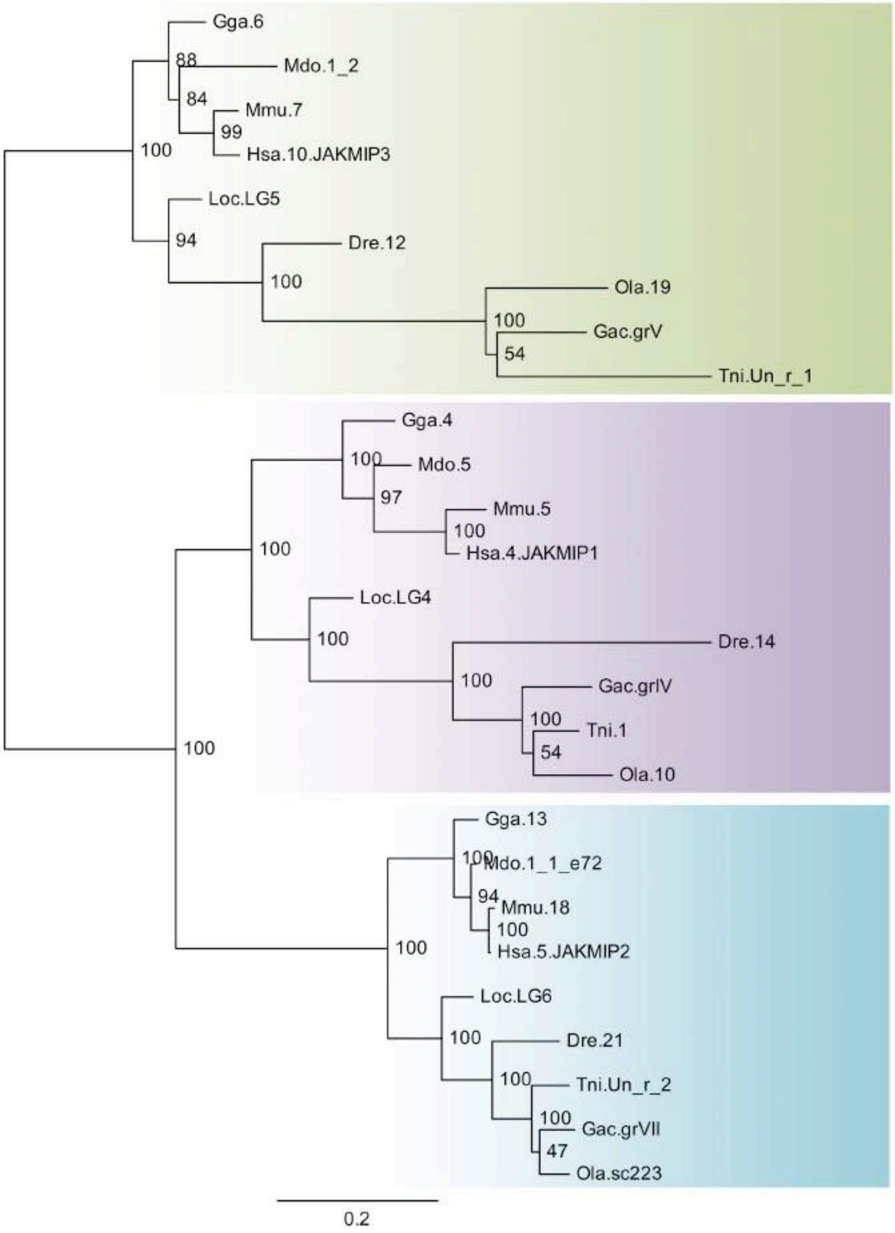

Supplementary figure S8: Phylogenetic maximum likelihood tree of the JAKMIP protein family using 100 bootstrap and the JTT amino acid substitution model. The tree was midpoint-rooted. Due to the lack of invertebrate sequences for relative dating, it is not possible to say when the duplications that resulted in the three vertebrate clusters occurred. However, the presence of three clusters is possible if the family duplicated in 2R. Species abbreviations are summarised in table 2, the number following the species abbreviation is either the chromosome or scaffold number or transcript id/accession number.

S9 – SH3TC

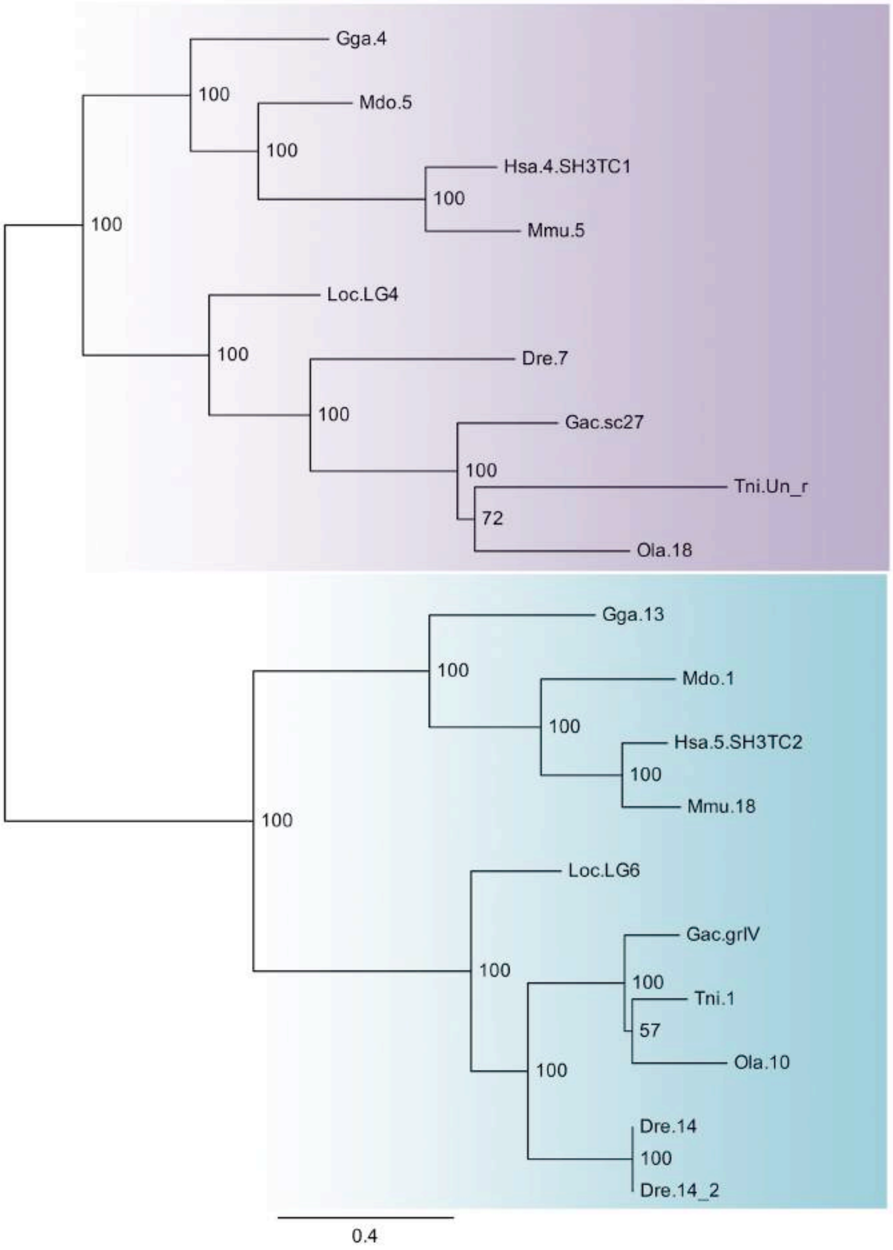

Supplementary figure S9: Phylogenetic maximum likelihood tree of the SH3TC protein family using 100 bootstrap and the JTT amino acid substitution model. The tree was midpoint-rooted. Due to the lack of invertebrate sequences for relative dating it is not possible to say when the duplications that resulted in the two vertebrate clusters occurred. However, the presence of two clusters is possible if the family duplicated in 2R. Species abbreviations are summarised in table 2, the number following the species abbreviation is either the chromosome or scaffold number or transcript id/accession number.

S10

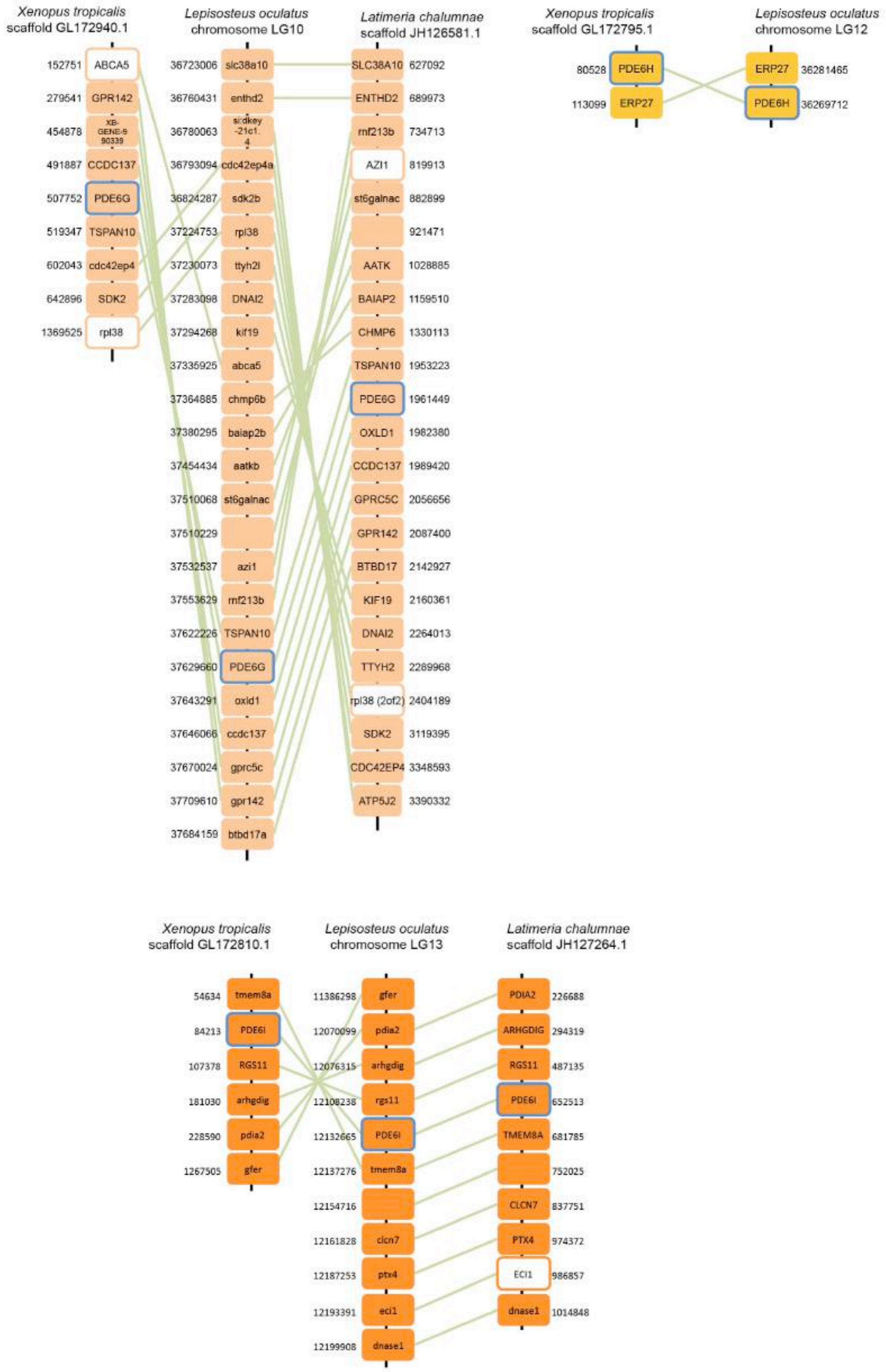

Supplementary figure S10: Genes present within 1 Mb up and downstream of spotted gar PDE6 inhibitory subunit genes with orthologs on a PDE6 inhibitory subunit gene carrying scaffold in Western clawed frog and coelacanth. Boxes are coloured based on the Ensembl orthology confidence where solid boxes are 1 and white boxes are 0.

S11

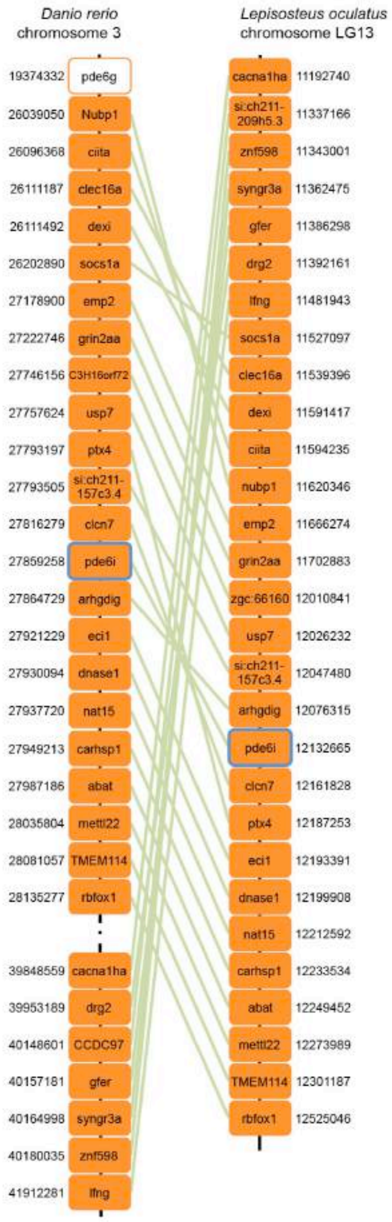

Supplementary figure S11: Genes present within 1 Mb up and downstream of spotted gar PDE6 inhibitory subunit genes with orthologs on a PDE6 inhibitory subunit gene carrying chromosome in zebrafish. Boxes are coloured based on the Ensembl orthology confidence where solid boxes are 1 and white boxes are 0.

S12

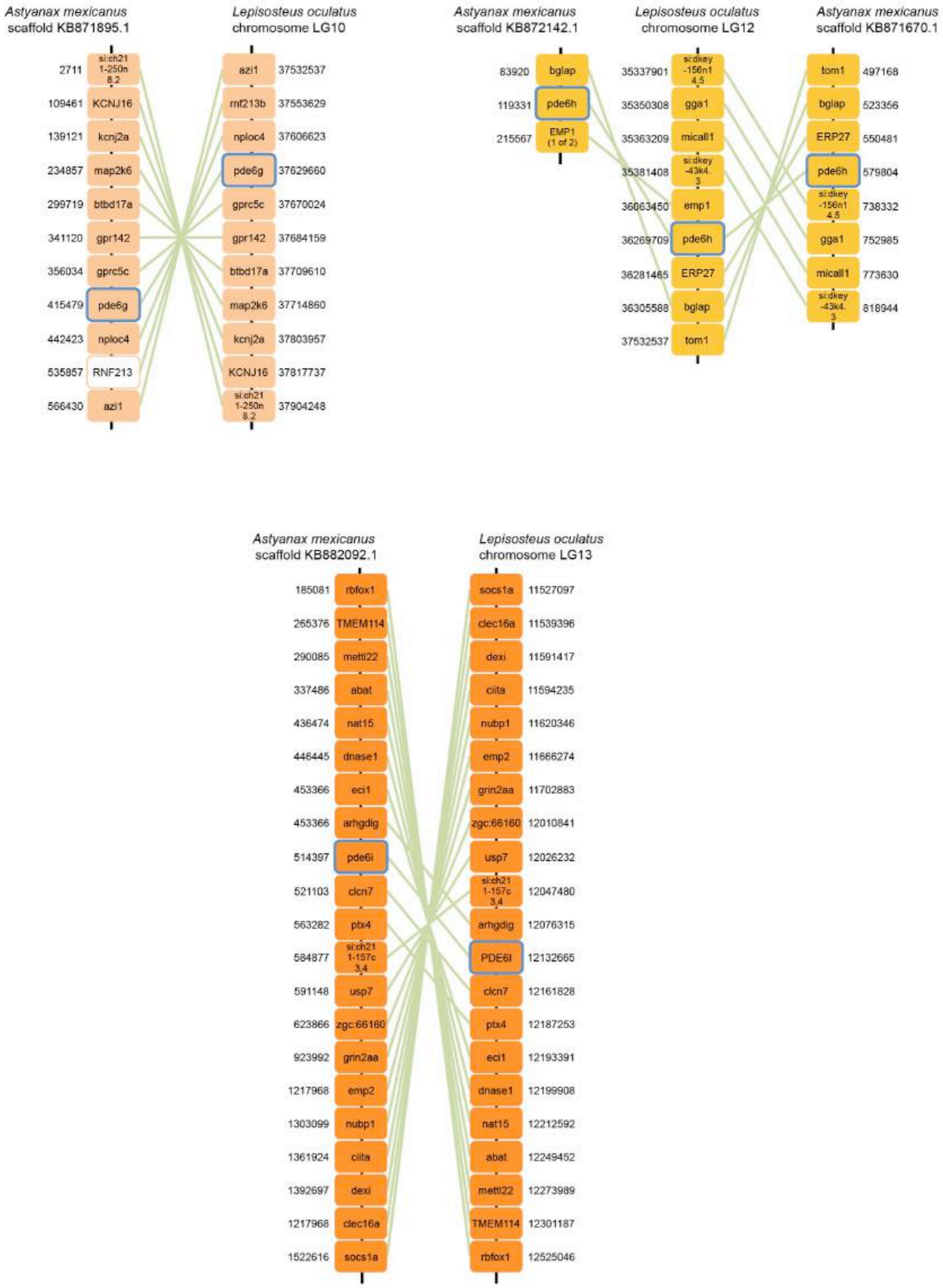

Supplementary figure S12: Genes present within 1 Mb up and downstream of spotted gar PDE6 inhibitory subunit genes with orthologs on a PDE6 inhibitory subunit gene carrying scaffold in Mexican cave tetra. Boxes are coloured based on the Ensembl orthology confidence where solid boxes are 1 and white boxes are 0.

S13

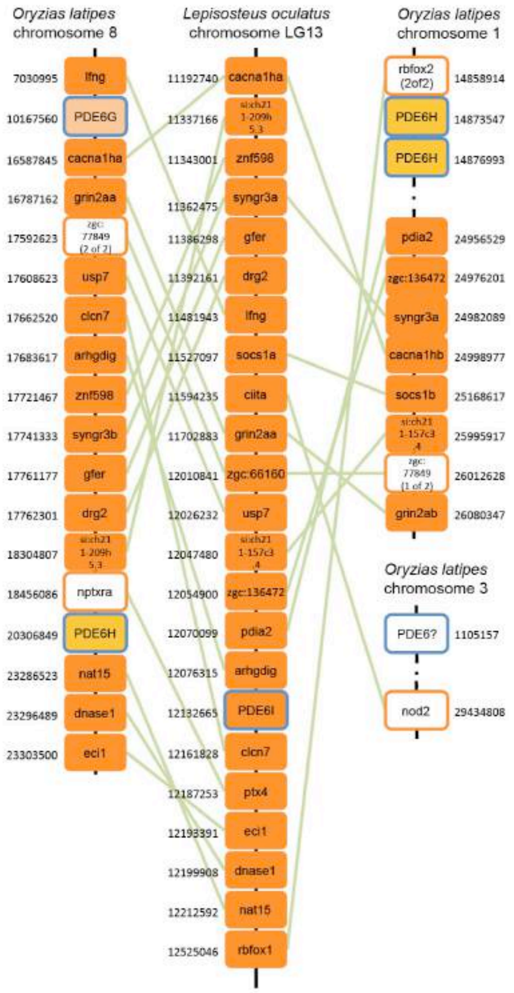

Supplementary figure S13: Genes present within 1 Mb up and downstream of spotted gar PDE6 inhibitory subunit genes with orthologs on a PDE6 inhibitory subunit gene carrying chromosome in Medaka. Boxes are coloured based on the Ensembl orthology confidence where solid boxes are 1 and white boxes are 0.

S14

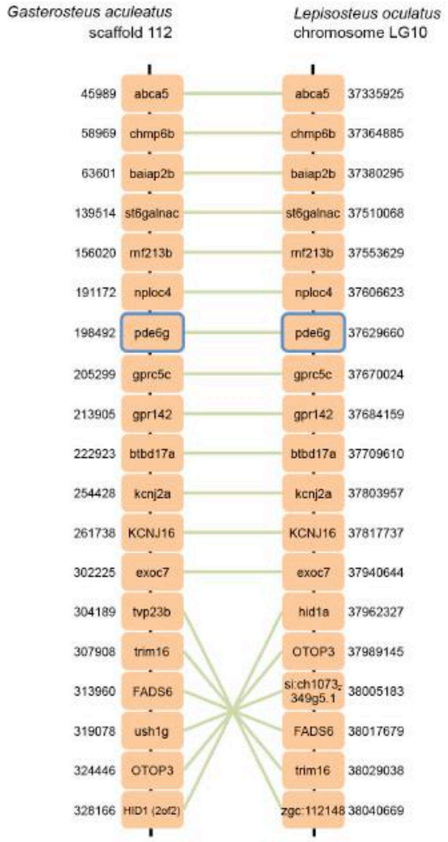

Supplementary figure S14: Genes present within 1 Mb up and downstream of spotted gar PDE6 inhibitory subunit genes with orthologs on a PDE6 inhibitory subunit gene carrying scaffold in three-spined stickleback. Boxes are coloured based on the Ensembl orthology confidence where solid boxes are 1 and white boxes are 0.

S15

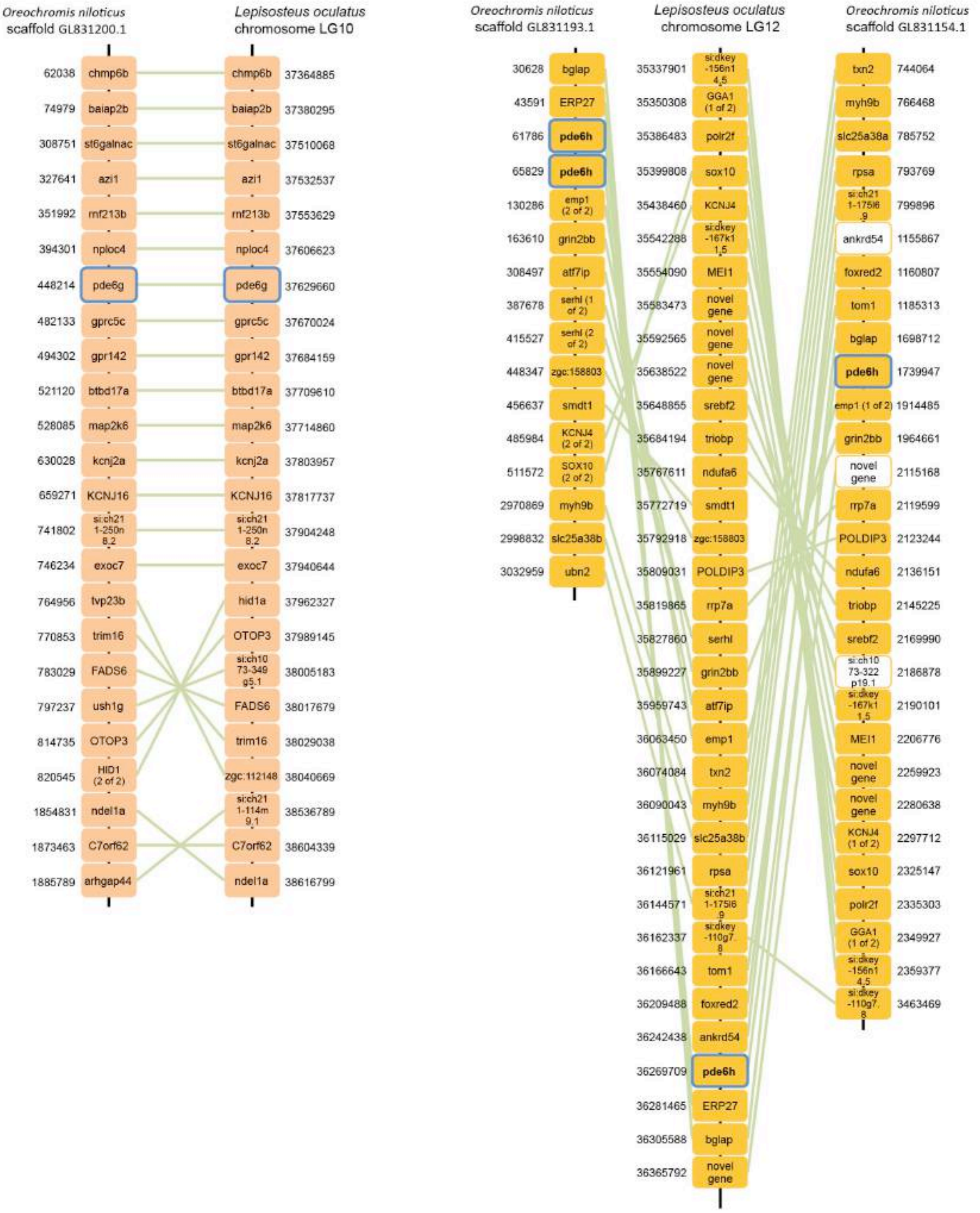

Supplementary figure S15: Genes present within 1 Mb up and downstream of spotted gar PDE6 inhibitory subunit genes with orthologs on a PDE6 inhibitory subunit gene carrying scaffold in Nile tilapia. Boxes are coloured based on the Ensembl orthology confidence where solid boxes are 1 and white boxes are 0.

S16

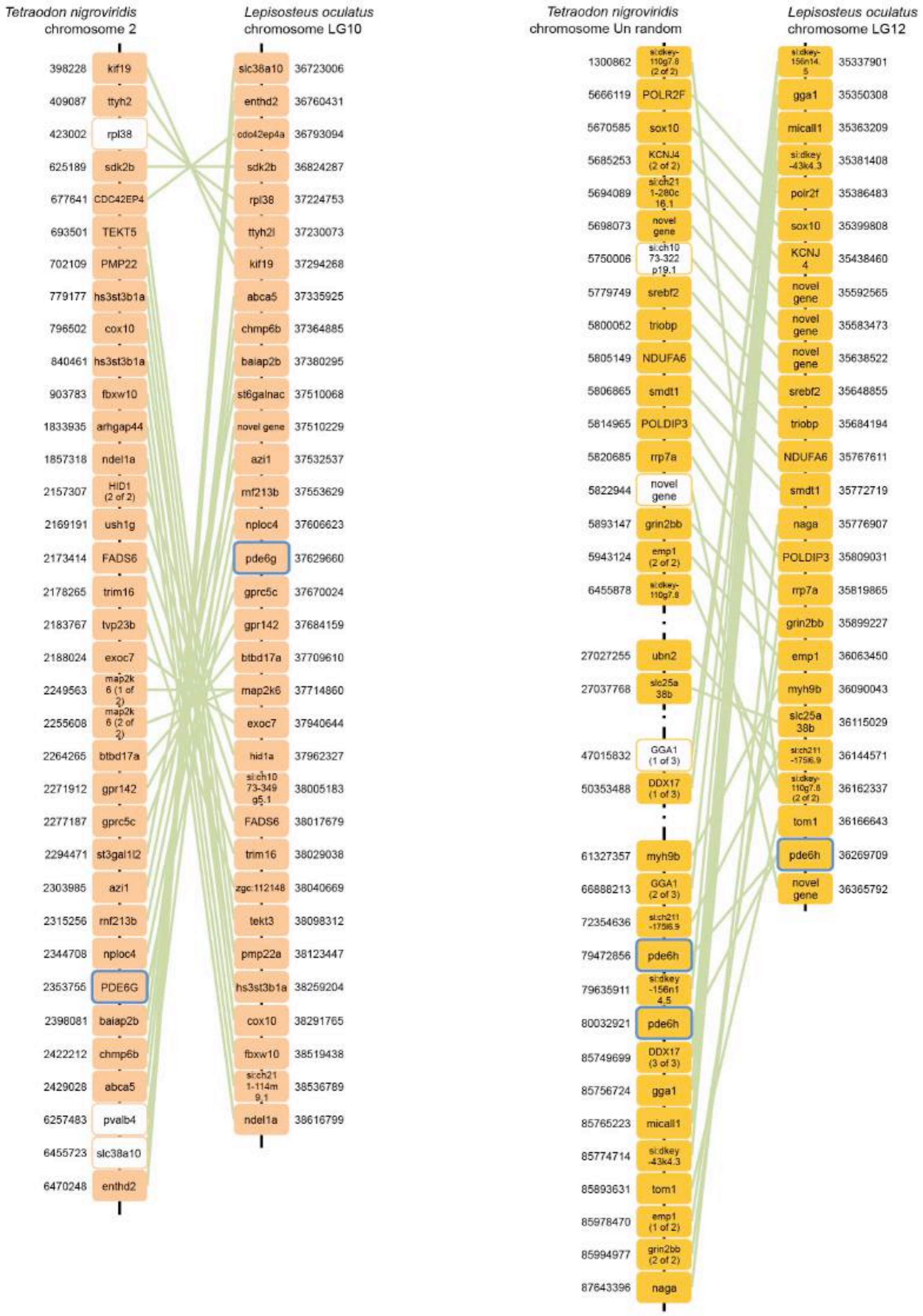

Supplementary figure S16: Genes present within 1 Mb up and downstream of spotted gar PDE6 inhibitory subunit genes with orthologs on a PDE6 inhibitory subunit gene carrying chromosome in green spotted pufferfish. Boxes are coloured based on the Ensembl orthology confidence where solid boxes are 1 and white boxes are 0.

S17

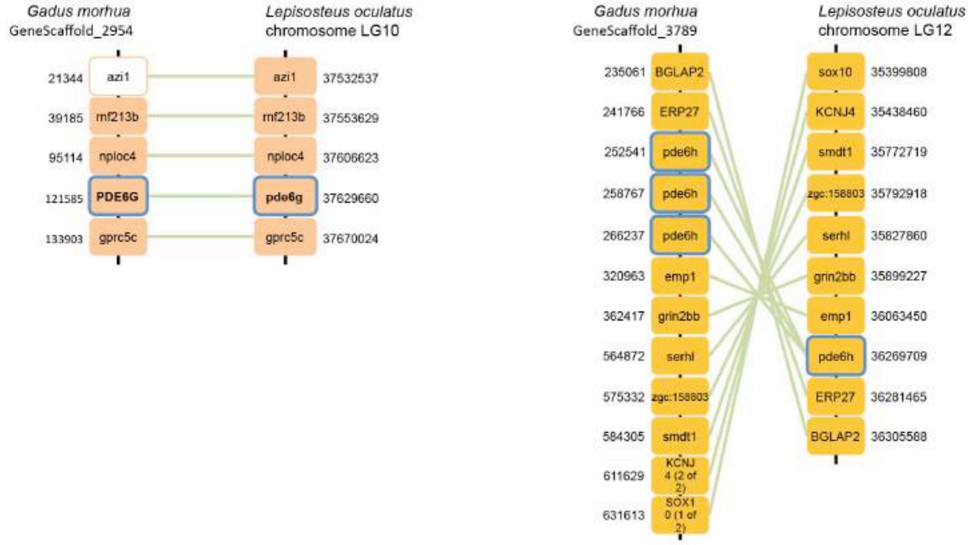

Supplementary figure S17: Genes present within 1 Mb up and downstream of spotted gar PDE6 inhibitory subunit genes with orthologs on a PDE6 inhibitory subunit gene carrying scaffold in Atlantic cod. Boxes are coloured based on the Ensembl orthology confidence where solid boxes are 1 and white boxes are 0.

S18

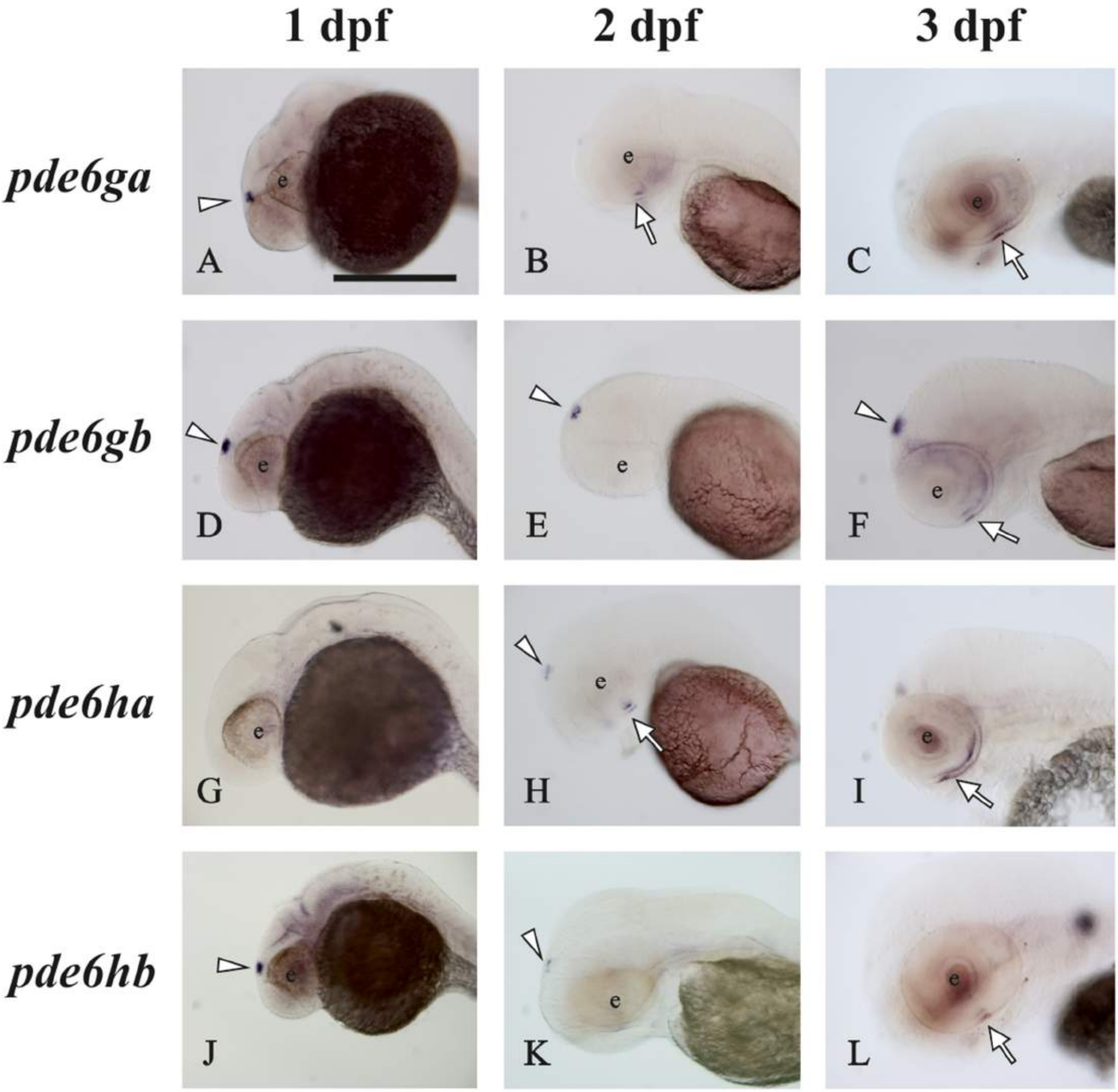

Supplementary figure S1: Onset of the PDE6 inhibitory subunit genes expression during zebrafish development. Photomicrographs of WISH experiments on 1-3 dpf AB zebrafish embryos showing staining for *pde6ga*, *pde6gb*, *pde6ha* and *pde6hb* genes. Staining in the pineal complex (arrowheads) could be first observed at 1 dpf for *pde6ga* (**A**), *pde6gb* (**D**) and *pde6hb* (**J**) while at 2 dpf for *pde6ha* (**H**). This expression is not transient, hence can be observed also in adults (see figure 3). The expression in the retina starts in the ventral side (arrows) and is delayed until around 2 dpf. For *pde6ga* (**B**) and *pde6ha* (**H**), the first stained cells can be observed at 2 dpf (48 hpf exactly), while the staining for *pde6ga* and *pde6hb* is not observed at 2 dpf (**E**, **K**) but is almost in the entire retina at 3 dpf (**F**, **L**). This suggests, as is the case for *transducins* (Lagman et al. 2015), an unsynchronised onset, between 48 and 52 hpf. Rostral is to the left, "e" indicates the eyes. Scale bars: 50 µm.

Table S1. Identified neighbouring gene families in the chromosomal regions housing the PDE6 catalytic subunit genes.

Symbol	Description	Comments	Ensembl. Family ID	Outgroup used as root[1]
ABLIM	*ACTIN BINDING LIM ABLIM ACTIN BINDING LIM FAMILY MEMBER*		ENSFM00250000000459	
AFAP	*ACTIN FILAMENT ASSOCIATED 1 1 AFAP1 1*		ENSFM00570000851128	
CP	*CARBOXYPEPTIDASE*	Removed from analysis due to complex topology	ENSFM00250000000555	
DPYS	*DIHYDROPYRIMIDINASE RELATED DRP COLLAPSIN RESPONSE MEDIATOR CRMP*	Mouse sequences were left out of the analysis due to the multitude of members in the family	ENSFM00250000000643	*Dme*
JAKMIP	*JANUS KINASE AND MICROTUBULE INTERACTING 1 MULTIPLE ALPHA HELICES AND RNA LINKER 1 MARLIN 1*		ENSFM00250000001480	
PPP2R2	*SERINE/THREONINE PHOSPHATASE 2A 55 KDA REGULATORY SUBUNIT B PP2A SUBUNIT B B55 PP2A SUBUNIT B PR55 PP2A SUBUNIT B R2 PP2A SUBUNIT B*		ENSFM00250000000829	*Dme*
SH3TC	*SH3 DOMAIN AND TETRATRICOPEPTIDE REPEAT CONTAINING*		ENSFM00250000003090	
SLC26A	*SOLUTE CARRIER FAMILY 26 MEMBER*	Removed from analysis due to complex topology	ENSFM00250000000278	
STK32	*SERINE/THREONINE KINASE EC_2.7.11.1*		ENSFM00260000050596	*Dme*
TBC1D	*TBC1 DOMAIN FAMILY MEMBER*		ENSFM00250000002496	*Cin*
ZNF	*ZINC FINGER*	Removed from analysis due to complex topology	ENSFM00250000000002	

[1]The Ensembl protein families that lack invertebrate family members were left unrooted in the phylogentic analyses and visualzed as midpoint-rooted in supplementary figs. S2-S9.

Table S1: The table lists the identified neighbouring gene families with members in at least two of three regions spanning approximately 10 mega base pairs up stream and downstream of the human PDE6 catalytic subunit genes.

Table S2: Percentage identity between zebrafish PDE6 inhibitory subunit gene 3′UTR sequences

Gene	*pde6ga*	*pde6gb*	*pde6ha*	*pde6hb*	*pde6i*
pde6ga	-	47.74	41.16	43.86	51.72
pde6gb		-	53.19	48.18	47.85
pde6ha			-	58.91	51.60
pde6hb				-	47.58
pde6i					-

Table S2: Nucleotide sequence identity from pair-wise sequence alignments between the zebrafish PDE6 inhibitory subunit 3′UTR sequences. The alignments were done in JalView 2.7.

Table S3. Results from one-way ANOVA of the relative expression for each PDE6 subunit gene between at six time-points during 24 hours

pde6a

Time	08:00	12:00	17:00	20:00	24:00	04:00
08:00	-	ns	ns	ns	ns	ns
12:00		-	ns	ns	ns	ns
17:00			-	ns	ns	ns
20:00				-	*	ns
24:00					-	*
04:00						-

pde6b

Time	08:00	12:00	17:00	20:00	24:00	04:00
08:00	-	ns	ns	ns	ns	*
12:00		-	ns	ns	ns	ns
17:00			-	ns	ns	ns
20:00				-	ns	ns
24:00					-	ns
04:00						-

pde6c

Time	08:00	12:00	17:00	20:00	24:00	04:00
08:00	-	*	***	***	**	**
12:00		-	ns	ns	ns	ns
17:00			-	ns	ns	ns
20:00				-	ns	ns
24:00					-	ns
04:00						-

pde6ga

Time	08:00	12:00	17:00	20:00	24:00	04:00
08:00	-	***	***	***	***	**
12:00		-	ns	ns	ns	***
17:00			-	ns	ns	***
20:00				-	ns	***
24:00					-	*
04:00						-

pde6gb

Time	08:00	12:00	17:00	20:00	24:00	04:00
08:00	-	ns	ns	ns	ns	ns
12:00		-	ns	ns	*	ns
17:00			-	ns	ns	ns
20:00				-	*	ns
24:00					-	ns
04:00						-

pde6ha

Time	08:00	12:00	17:00	20:00	24:00	04:00
08:00	-	ns	ns	ns	*	**
12:00		-	ns	*	**	***
17:00			-	*	**	***
20:00				-	ns	ns
24:00					-	ns
04:00						-

pde6hb						
Time	08:00	12:00	17:00	20:00	24:00	04:00
08:00	-	*	ns	***	***	ns
12:00		-	ns	ns	ns	ns
17:00			-	**	*	ns
20:00				-	ns	**
24:00					-	*
04:00						-

Table S3: One-Way ANOVA with Tukey's Multiple Comparison post-hoc test results for the expression levels relative to the geometric mean of *actb1* and *tuba1b* of the different PDE6 subunit genes, both catalytic and inhibitory. The statistics were done in GraphPad Prism 5.00. Ns: non-significant difference.
