# Supplementary material for: Evolution and expression of the phosphodiesterase 6 genes unveils vertebrate novelty to control photosensitivity
Source: BMC Evol Biol. 2016 Jun 13;16:124. doi: 10.1186/s12862-016-0695-z (PMC4906994; doi:10.1186/s12862-016-0695-z)
Supplement: Additional file 4: — Multiple sequence alignment in FASTA format for the amino acid sequences of the identified PDE6 inhibitory subunit genes. The alignment was done using ClustalO in the Seaview 4.5.3 program. Sequence names are provided in Additional file 2. (RTF 9 kb) [file 12862_2016_695_MOESM4_ESM.rtf]

>Gac.grIX_1
---------------------M-------ADVAV----------------
---------PADRKA-PPKFKQRTARTFKSKAPKPGQKGFGDDIPGMEGL
GTDITVVCPWEAFGDMELSDLAKYGIV
>Ola.1_1
---------------------M-------ADVAT----------------
---------PADRKA-PPKFKQRTTRTFKSKAPKPGQKGFGDDIPGMEGL
GTDITVVCPWEAFGDMELSDLAKYGIV
>Oni.GL831193.1_1
---------------------M-------ADVAT----------------
---------PADKKA-PPKFKQRAARTFKSKAPKPGQKGFGDDIPGMEGL
GTDFTVVCPWEAFGDMELSDLAKYGIV
>Oni.GL831193.1_2
---------------------M-------ADAPA----------------
---------PADKKA-PPKFKQRTARTFKSKAPKPGQKGFGDDIPGMEGL
GTDFTVVCPWEAFGDMELSDLAKYGIV
>Ola.1_2
---------------------M-------ADAAVPT--------------
---------PADKKA-PPKFKQRTTRTFKSKAPKPGQKGFGDDIPGMEGL
GTDITVVCPWEAFGDMELSDLAKYGIV
>Tni.Un_r_1
---------------------M-------SDTAVDA--------------
---------PVDKKA-PPKFXQRTARTFKSKAPKPGQKGFGDDIPGMEGL
GTDITVVCPWEAFGDMELSDLAKYGIV
>Ame.KB871670.1
-----------------------------MDVAT----------------
---------PAEKKA-PPKFKARTTRTFKSKAPKPGQKGFGDDIPGMEGL
GTDFTVVCPWEAFGDMELSDLAKFGIV
>Tni.Un_r_2
---------------------M-------ADVAT----------------
---------PADEKV-PPXFKQRTA-TFRSRAPRPGQKGFRDDIPGMEGL
GTDTTVICPWEAFGDMELSNLAKYRII
>Gmo.gs3789_1
-----------------------------MDIAVPA--------------
---------AADKKA-APRFKQRAARTFKSKAPKPGQKGFGDDIPGMEGL
GTDITVVCPWEAYGDLELGDLAKYGIV
>Gmo.gs3789_3
-----------------------------MDIAVPA--------------
---------AADKKA-APRFKQRAARTFKSKAPKPGQKGFGDDIPGMEGL
GTDITVVCPWEAYGDLELGDLAKYGIV
>Ga.grIX_2
---------------------M-------SDEAAGN--------------
---------LAEKKA-APKFKQRAMRTFKSKAPKPGQKGFGDDIPGMEGL
GTDITVVCPWEAFGDMELSDLAKYGIV
>Gmo.c43845
--------------------------------KMAD--------------
---------PAEKKA-PPKFKQRSTRTFKSRAPKPGQKGFGDDIPGMEGL
GTDITVVCPWEAYGDLELGDLAKYGIV
>Gmo.gs3789_2
---------------------------------MAD--------------
---------PAEKKA-PPKFKQRSARTFKSRAPKPGQKGFGDDIPGMEGL
GTDITVVCPWEAYGDLELGDLAKYGIV
>Dre.NP_957131.1
----------------MNSAPP-------AGSALATP-------------
---AATTGPTTPKKG-PPKFKQRQTRTFKSKAPKPGQKGFGDDIPGMEGL
GTDFTVVCPWEAFGDMELSDLAKYGII
>Gmo.gs1588
----------------MNAAPP-------AGSALAA--------------
---NTTTGPTTPKKG-PPKFKQRQTRTFKSKAPKPGQKGFGDDIPGMEGL
GTDITVVCPWEAFGDMELSDLAKYGII
>Gac.grXI
----------------MNAAPP-------AGSALGP--------------
---TATIAPTTPKKG-PPKFKQRQARTFKSKAPKPGQKGFGDDIPGMEGL
GTDITVVCPWEAFGDMELSDLAKYGII
>Ola.8_1
----------------MNASPP-------AGSALAP--------------
---GGATGPTTPKKG-PPKFKQRQTRTFKSKAPKPGQKGFGDDIPGMEGL
GTDITVVCPWEAFGDMELSDLAKYGII
>Oni.GL831154.1
----------------MNASPP-------AGSALAP--------------
---SGATGPTTPKKG-PPKFKQRQTRTFKSKAPRPGQKGFGDDIPGMEGL
GTDFTVVCPWEAFGDMELSDLAKYGII
>Tni.1
----------------MNANPP-------AGSALAP--------------
---GGSTGPTTPKKG-PPKFKQRQTRTFKSKAPKPGQKGFGDDIPGMEGL
GTDITVVCPWEAFGDMELSDLAKYGII
>Ame.KB872142.1
----------------MNSAP--------ASSALAAP-------------
---AGGAGPTTPKKG-PPKFKQRQTRTFKSKAPKPGQKGFGDDIPGMEGL
GTDFTVVCPWEAFGDMELSDLAKYGII
>Dre.NP_957079.1
-----------------------------MDVAE----------------
---------PVEKRGGPPKFKQRTTRTFKSKAPKPGQKGFGDDIPGMEGL
GTDITVICPWEAFGDMELSDLAKYGIL
>Aca.XP_003221133.1
----------------MTESNP-------PPANLNT--------------
---DPATTGSSTPRKGPPKFKQRQTRQFKSKPPKKGVKGFGDDIPGMEGL
GTDITVICPWEAFSHLELHELAQFGII
>Gga.NP_989739.1
----------------MSEN---------PTTNLTT--------------
---GDAPTGPTTPRKGPPKFKQRQTRQFKSKPPKKGVKGFGDDIPGMEGL
GTDITVICPWEAFSHLELHELAQFGII
>Mdo.8
----------------MN-----------DNNVLAP--------------
---PVSGQGPTTPRKAPPKFKQRQTRQFKSKPPKKGVKGFGDDIPGMEGL
GTDITVICPWEAFSHLELHELAQFGII
>Mdo.Un
----------------MN-----------DNNVLAP--------------
---PVSGQGPTTPRKAPPKFKQRQTRQFKSKPPKKGVKG-----------
---------------------------
>Hsa.12.PDE6H
----------------MS-----------DNTTLPA--------------
---PASNQGPTTPRKGPPKFKQRQTRQFKSKPPKKGVKGFGDDIPGMEGL
GTDITVICPWEAFSHLELHELAQFGII
>Mmu.6
----------------MS-----------DSPSLSP--------------
---PAPSQGPTTPRKGPPKFKQRQTRQFKSKPPKKGVKGFGDDIPGMEGL
GTDITVICPWEAFSHLELHELAQFGII
>Lch.JH132110.1
----------------MNSSPP-------ASNTLTP--------------
---PNVANGPTTPRKGPPKFKQRQTRQFKSKPPKKGVKX-----------
---------------------------
>Xtr.GL172795.1
----------------MNSGSP-------ASSALAP--------------
---VNNSGGPTTPRKGPPKFKQRATRQFKSKPPKKGVKGITEVQHGHLFS
FADITVICPWEAFSHLELHELAQFGII
>Xtr.GL172780.1
----------------MNSGSP-------ASSALAP--------------
---VNNSGGPTTPRKGPPKFKQRATRQFKSKPPKKGVKGFGDDIPGMEGL
GTDITVICPWEAFSHLELHELAQFGII
>Pma.ABO16480.2
------------------MSEK-------TSNTLAP--------------
---PVTHTGPTTPKKGPPKFKQRATRQFKSKPPKPGVKGFGDEIPGMEGL
GTDITVICPWEAFSHLELHELAQYGIV
>Loc.LG12
----------------MNTQPP-------AGSALSA--------------
---PAPLDAGSTPKKGPPKFKQRQTRQFKSKPPKKGVKGFGDDIPGMEGL
GTDITVICPWEAFSHLELHELAQFGIV
>Aca.XP_003217086.1
----------------MSLEPP-------TPEVKSA--------------
---TKVTGGPVTPRKGPAKFKQRQTRQFKSKPPKKGIQGFGDDIPGMEGL
GTDITVICPWEAFSHLELHELAQYGII
>Gga.NP_989776.1
----------------MSLEPH-------KPELKSA--------------
---TRVTGGPATPRKGPPKFKQRQTRQFKSKPPKKGVQGFGDDIPGMEGL
GTDITVICPWEAFSHLELHELAQYGII
>Mdo.2
----------------MNLEPP-------KLEIRSA--------------
---TRVVGGPVTPRKGPPKFKQRQTRQFKSKPPKKGVQGFGDDIPGMEGL
GTDITVICPWEAFNHLELHELAQYGII
>Mmu.11
----------------MNLEPP-------KGEIRSA--------------
---TRVIGGPVTPRKGPPKFKQRQTRQFKSKPPKKGVQGFGDDIPGMEGL
GTDITVICPWEAFNHLELHELAQYGII
>Hsa.17.PDE6G
----------------MNLEPP-------KAEFRSA--------------
---TRVAGGPVTPRKGPPKFKQRQTRQFKSKPPKKGVQGFGDDIPGMEGL
GTDITVICPWEAFNHLELHELAQYGII
>Xtr.GL172940.1
----------------MNLEPA-------KAEIKSA--------------
---TRVTGGPATPRKGPPKFKQRQTRQFKSKPPKKGVQGFGDDIPGMEGL
GTDITVICPWEAFNHLELHELAQYGII
>Oni.GL831200.1
----------------MNLEPP-------KAEIKSA--------------
---TRVSGGPATPRKGPPKFKQRQTRQFKSKPPKKGVQGFGDDIPGMEGL
GTDITVICPWEAFNHLELHELAQYGII
>Ola.19
----------------MNLEPP-------KAEIKSA--------------
---TRVSGGPATPRKGPPKFKQRQTRQFKSKPPKKGVQGFGDDIPGMEGL
GTDITVICPWEAFSHLELHELAQYGII
>Tni.2
----------------MNLEP--------KAEIKSA--------------
---TRVSGGPATPRKGPPKFKQRQTRQFKSKPPKKGVQGFGDDIPGMEGL
GTDITVICPWEAFSHLELHELAQYGII
>Gac.sc112
----------------MNLELP-------KADIKSA--------------
---TRVSGGPATPRKGPPKFKQRQTRQFKSKPPKKGIQGFGDDIPGMEGL
GTDITVICPWEAFNHLELNELAKYGII
>Gmo.gs2954
----------------MNLEAP-------KAEIKSA--------------
---TRVSGGPATPRKGPPKFKQRQTRQFKSKPPKKGIQGFGDDIPGMEGL
GTDITVICPWEAFNHLELNELAKYGII
>Ame.KB871895.1
MTGDTEHIPPPIPVAALNVEQP-------KSDIKSA--------------
---TRVSGGPATPRKGPPKFKQRQTRQFKSKPPKKGIQGFGDDIPGMEGL
GTDITVICPWEAFNHLELHELAQYGII
>Lch.JH126581.1
-MEVQKTVNLEIP-KTVNLEVP-------KPEVKSA--------------
---TRVSGGPATPRKGPPKFKQRQTRQFKSKPPKKGIQGFGDDIPGMEGL
GTDITVICPWEAFSHLELHELAQYGII
>Loc.LG10
----------------MNLEPP-------KPEIKSA--------------
---TRVSGGPATPRKGPPKFKQRQTRQFKSKPPKKGIQGFGDDIPGMEGL
GTDITVICPWEAFSHLELHELAQYGII
>Dre.NP_997964.1
----------------MNLEPP-------KPEIKSA--------------
---TRVTGGPATPRKGPPKFKQRQTRQFKSKPPKKGIQGFGDDIPGMEGL
GTDITVICPWEAFNHLELHELAQYGII
>Pma.ABO64650.1
----------------MNLATANT-----SGALMAP--------------
---TKVSGGPATPRRGPSKFKQRQTRQFKSKPPKKGIQGFGDDIPGMEGL
GTDITVICPWEAFNHLELHELAQYGIL
>Xtr.GL172821.1
--------------------------------------------------
--------------------------------------XFGDDIPGMEGL
GTDITVICPWEAFNHLELHELAQYGII
>Lch.JH127264.1
----------------MNVAIH-F-----SGNPITP--------------
---TRVTGGPATPRKDPPKFKQRQTRQFKSKPPKKGIQGFGDDIPGMEGL
GTDITVICPWEAFSQLELHELAQYGII
>Loc.LG13
----------------MNISV----------NPTTP--------------
---TKVTGGPTTPRKGPPKFKQRQTRQFKSKPPKKGVKGFGDDIPGMEGL
GTDITVICPWEAFSHLELHELAQFGII
>Ola.8_2
--------------MNLEVAKPEA-----KSGSKAP--------------
---LRAT-GPGSPHKGPPKFKQRNTRTFKSKPPKRGVIGFGEEIPGMEGL
GTDITVICPWEAYSHLELHELAQYGII
>Oni.GL831217.1
--------------MNLEVAKPEG-----KTGNKAP--------------
---LRAT-APGSPRKSAPKFKQRNTRQFKSKPPKRGVIGFGEEIPGMEGL
GTDITVICPWEAYSHLELHELAQYGII
>Dre.NP_001153298.1
----------------MDVSKP-------KSTSKGA--------------
---TRAT-GPGSPHKGPPKFKQRSTRQFKSKPPKKGVIGFGEEIPGMEGL
GTDFNVICPWEAYSHLELHELAQYGII
>Ame.KB882092.1
----------------MSSTSS-------SSSSSSSSSSSSSSIGVSDVS
SCPTRAINGPTTPRKGPPKFKQRMTRHFKSKPPKKGVKGFGDEIPGMEGL
GNDYTVVCPWEAFSHLELHELAQYGII
>Dre.XP_003198167.2
----------------MNSRNC-------NSLNNSQ--------------
---VRARAGTTTPRSGPPEFKQGMIRQFKSKTPKKGVKGSGDEIPGMDGL
GTDTTVVCPWEAFSHLELNQLAQYGII
>Xtr.GL172810.1
----------------MGKSTDVLDKKRKETEVKAAEEVNVPSENIG---
---ATSSQPPARQRKGP-KFKQRGSKEFKSKPPKKGVIGF-EEIPGMEGL
GQDITVVCPWEAYSHLELHELAQYGII
>Ola.3
-----------------------------MNAAAAA--------------
--------AGE-DKAASSEFQQKESRQFKSKAPKPGQKRFDSSLPGMESF
D-DSAVVCPWEEFGDANLSELAQFGII
>Oni.GL831252.1
-----------------------------MNATGAE--------------
--------PAEGSKPAPPKFKQKGSRQFKSKAPKPGQKGFDNDVPGMEEL
G-DSAVVCPWEAFGDMELSDLAQFGVV
